# Germline and somatic imprinting in the nonhuman primate highlights species differences in oocyte methylation

**DOI:** 10.1101/gr.183301.114

**Published:** 2015-05

**Authors:** Clara Y. Cheong, Keefe Chng, Shilen Ng, Siew Boom Chew, Louiza Chan, Anne C. Ferguson-Smith

**Affiliations:** 1 Growth, Development and Metabolism Program, Singapore Institute for Clinical Sciences, Agency for Science, Technology and Research (A-STAR), Singapore 117609;; 2 Department of Genetics, University of Cambridge, Cambridge CB2 3EH, United Kingdom

## Abstract

Genomic imprinting is an epigenetic mechanism resulting in parental allele-specific gene expression. Defects in normal imprinting are found in cancer, assisted reproductive technologies, and several human syndromes. In mouse models, germline-derived DNA methylation is shown to regulate imprinting. Though imprinting is largely conserved between mammals, species- and tissue-specific domains of imprinted expression exist. Using the cynomolgus macaque (*Macaca fascicularis*) to assess primate-specific imprinting, we present a comprehensive view of tissue-specific imprinted expression and DNA methylation at established imprinted gene clusters. For example, like mouse and unlike human, macaque *IGF2R* is consistently imprinted, and the *PLAGL1, INPP5F* transcript variant 2, and *PEG3* imprinting control regions are not methylated in the macaque germline but acquire this post-fertilization. Methylome data from human early embryos appear to support this finding. These suggest fundamental differences in imprinting control mechanisms between primate species and rodents at some imprinted domains, with implications for our understanding of the epigenetic programming process in humans and its influence on disease.

Genomic imprinting is an epigenetically regulated process resulting in gene expression from specific parental alleles. Many imprinted genes are clustered and feature both protein-coding and noncoding RNA genes (Edwards and Ferguson-Smith 2007). In mouse, differential DNA methylation at CpG-rich imprinting control regions (ICRs) is first established in gametogenesis, along with other methylation marks, and depends on the presence of DNA methyltransferases (DNMTs) (Li et al. 1993; Okano et al. 1999; Li and Sasaki 2011). During preimplantation development, protection from demethylation is essential at imprints (Li et al. 2008; Hanna and Kelsey 2014), and subsequently, additional differentially methylated regions (DMRs) can become established in response to the germline DMR (Kafri et al. 1992; Brandeis et al. 1993a,b).

Imprinted genes are involved in both pre- and post-natal growth, and metabolic and cognitive processes (Ferguson-Smith 2011; Cleaton et al. 2014). In humans, aberrant imprinting is responsible for certain developmental disorders with parental origin effects (Weksberg et al. 2003; Gicquel et al. 2005), while perturbed imprinting is regularly reported in cancers (Uribe-Lewis et al. 2011). More recently, the increased incidence of imprinting defects in infants conceived through assisted reproduction techniques emphasizes the importance of imprinting epigenetics from a very early developmental time point (Grace and Sinclair 2009).

Comparative analysis of imprinting between eu-, meta- and prototherian mammals suggests that imprinting arose relatively recently at most loci—only a few imprinted genes in Eutherians are also imprinted in marsupials, while no imprinting has been reported in the egg-laying monotreme mammals to date (Killian et al. 2000; Edwards et al. 2008; Smits et al. 2008; Renfree et al. 2009a,b). While the mouse is an informative proxy for human imprinted gene regulation, not all loci show conserved imprinting, notably in the placenta (Tycko and Morison 2002; Morison et al. 2005). Distinct differences in placental evolution, physiology, and reproductive biology of the primate and murine groups may be responsible. In contrast to an evolutionary distance of 75 million years between mouse and human, the macaque diverged 25 million years ago from human and shares many physiological similarities with humans. The added availability of the macaque genome has made this nonhuman primate a useful model for understanding recent genomic evolutionary changes (Waterston et al. 2002; Rhesus Macaque Genome Sequencing and Analysis Consortium et al. 2007; Yan et al. 2011), with further potential for understanding the evolution of epigenetic mechanisms.

In order to explore the evolution of imprinting in the primate, we surveyed established imprinted gene clusters for the conservation of imprinted gene expression and DNA methylation in the nonhuman primate, cynomolgus macaque (*Macaca fascicularis*). The closely related cynomolgus and rhesus (*Macaca mulatta*) macaques are 99.6% similar, estimated to diverge by only ∼2 million years, and share much genomic structure and similarity, both with each other and with the human genome (Hayasaka et al. 1996; Osada et al. 2008). As such, both are widely used in preclinical studies and are useful models for accessing tissues otherwise limited in human research (Bourne 1975).

Here, we provide the most comprehensive survey of imprinting in the nonhuman primate to date and investigate the conservation of primary and secondary DMRs between primates and rodents. Our findings suggest that aspects of imprinting control may differ between rodent and macaque, with important implications for epigenetic programming in normal development and disease.

## Results

### Conservation of allelic expression at imprinted loci in macaque

We examined somatic and extraembryonic tissues for allelic expression at a total of 32 genes known to be imprinted in either human or mouse. Macaque genomic regions analyzed were identified by orthology to known human imprinted genes, since the sequence identity between human and macaque is ∼93% (Rhesus Macaque Genome Sequencing and Analysis Consortium et al. 2007), enabling reference gene mapping and the discovery of novel polymorphisms required for allele-specific expression analysis.

#### Paternally imprinted genes

**Figure 1.**
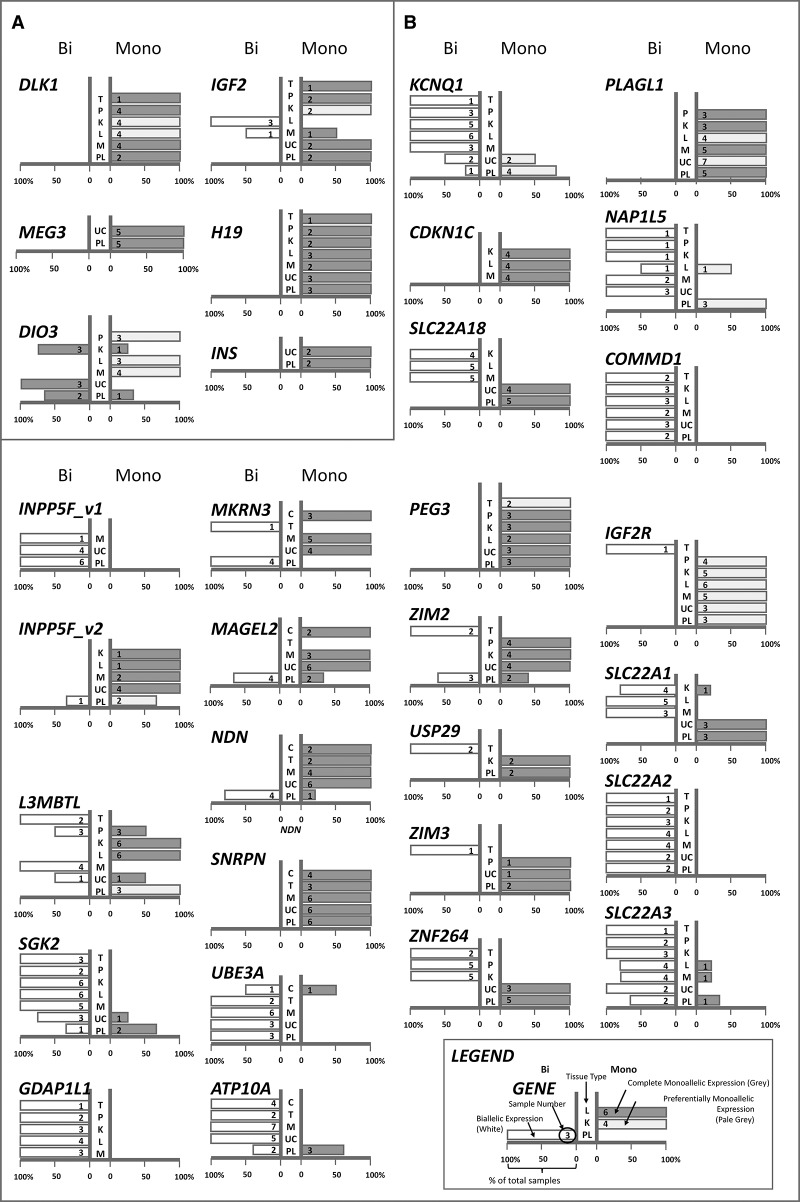
Imprinted expression profile in tissues. Individual charts show allelic expression for each gene across the main tissue types examined. (*A*) Paternally imprinted gene clusters. (*B*) Maternally imprinted gene clusters. The length of each bar represents the percentage of total samples adhering to either expression pattern—biallelic (*left*) or monoallelic (*right*). A further distinction is made between complete and preferential monoallelic expression at each gene: complete monoallelic expression (gray) and preferential monoallelic expression (pale gray). See Supplemental Figure 2 for quantitative pyrosequencing and capillary sequencing data. The number of samples analyzed is shown in each bar, and tissue type is listed in the central column of each chart. (C) Cerebellum, (T) Testes, (P) pancreas, (K) kidney, (L) liver, (M) muscle, (UC) umbilical cord, (PL) placenta. These data are also tabulated in Supplemental Table 1, with additional data available for select genes/tissues.

In mouse, the *Igf2-H19* and *Dlk1-Dio3* domains are controlled by paternal-specific germline methylation imprints at their intergenic ICRs (Kobayashi et al. 2000; de la Puente et al. 2002; Takada et al. 2002; Gabory et al. 2006; Cai and Cullen 2007). Consistent with neonatal rhesus tissues and ES cells, *IGF2* and *H19* are monoallelically expressed in all cynomolgus extraembryonic tissues analyzed (Fujimoto et al. 2005, 2006). *H19* expression is also consistently monoallelic in all somatic tissues tested, although the corresponding tissue imprinting of *IGF2* is somewhat relaxed, particularly in liver and skeletal muscle (Fig. 1A; Supplemental Table 1). Given the significance of *IGF2* in fetal growth and placental development (Han and Carter 2000; Constancia et al. 2002), it is perhaps expected that imprinted expression is most robust in placenta. Post early development, the functional role of *IGF2* is less clear, with relaxed biallelic imprinting reported in some somatic tissues of the macaque (Fig. 1A) and human (Davies et al. 2007; Frost et al. 2010). In addition, *INS*, a more distal gene, also shows consistent allele-specific expression in extraembryonic tissues (Fig. 1A).

The *DLK1* locus is another cluster with known paternal inheritance of methylation imprints and is expressed at similar developmental stages as the *IGF2-H19* locus (Takada et al. 2000, 2002). *DLK1* expression in the cynomolgus is fully monoallelic in placenta and some adult tissue (skeletal muscle, pancreas, testes). In adult kidney and liver, *DLK1* is preferentially expressed monoallelically, suggestive of incomplete or cell-specific imprinting. Placenta and umbilical cord samples at the adjacent *MEG3* (also known as *GTL2*) also show monoallelic expression; no somatic tissue samples were informative at this locus. Mouse *Dio3* is known to be preferentially expressed from the paternal allele in embryonic tissue but biallelically expressed in the placenta (Hernandez et al. 2002; Lin et al. 2007), and maternal deletion of the *Dlk1* ICR (intergenic DMR; IG-DMR) influences *Dio3* imprinting despite its physical distance from the ICR (Lin et al. 2007). In our macaque samples tested, imprinted expression at *DIO3* appears to be preferentially monoallelic in most somatic tissues and preferentially biallelic in placenta (Fig. 1A).

#### Maternally imprinted genes

Adjacent to the *IGF2-H19* cluster, the *KCNQ1* locus also retains its gene order and chromosomal syntenic homology between human, macaque, and mouse (Supplemental Fig. 3). Macaque *KCNQ1* and *SLC22A18* are largely monoallelically expressed in placenta, with a number of individuals showing preferential but incomplete monoallelic expression at *KCNQ1. KCNQ1* expression was, however, biallelic in adult tissues (Fig. 1B). In mouse, imprinted expression of *Kcnq1* is seen in embryos, but not in adult mice or humans. This embryonic stage-specific expression may also be true in primates and could account for the partial imprinting seen in term extraembryonic tissues (Lee et al. 1997; Caspary et al. 1998; Gould and Pfeifer 1998). Published findings on human *SLC22A18* suggest that polymorphic imprinting is evident in adult liver and kidney (Dao et al. 1998; Gallagher et al. 2006), though the population frequency of this occurrence is unknown. Our analysis of *SLC22A18* in macaques in these same tissues showed consistent biallelic expression (Fig. 1B).

The *KCNQ1* locus further highlights that gene-ICR proximity alone does not determine imprinted expression. *CDKN1C*, a cyclin-dependent kinase inhibitor, is positioned downstream from the intronic KvDMR and showed monoallelic expression in cynomolgus adult tissues. No informative extraembryonic tissues were available. In mouse, *Cdkn1c* has a somatic promoter DMR which may contribute to stable monoallelic expression of this gene (Nowak et al. 2011), though we did not find evidence for this DMR in the primate (data not shown). Further examination of cynomolgus *CDKN1C* transcripts also revealed a novel transcript variant with no precedent in human or mouse *CDKN1C* (Supplemental Fig. 4; Nielsen et al. 2005). The genomic sequence of *CDKN1C* is conserved between rhesus, cynomolgus, and human, but alternative splicing results in a cynomolgus-specific transcript that, if translated, is only conserved at the cyclin-dependent kinase inhibitor domain (CDI, Pfam 02234).

The *IGF2R* cluster is well conserved across species and contains IGF2R, a receptor for the oppositely imprinted *IGF2*, and three related solute carriers, *SLC22A1*, *SLC22A2*, and *SLC22A3* (Smrzka et al. 1995). In mouse, the noncoding *Airn* RNA transcript antisense to *Igf2r* is necessary for imprinting. In mouse, despite its position between the imprinted *Igf2r*, *Slc22a2*, and *Slc22a3* genes, *Slc22a1* is not imprinted (Lyle et al. 2000; Sleutels et al. 2002, 2003). The imprinting of human *IGF2R* has been questioned—various sources have suggested either polymorphic imprinting at relatively high frequency or, conversely, no imprinting (Xu et al. 1993; Wutz et al. 1998; Killian et al. 2001). In a survey of *IGF2R* imprinting across phylogenetic orders, it was also suggested that *IGF2R* imprinting was absent from an ancestor of the Euarchonta order, a mammalian order that includes humans and other primates (Killian et al. 2001). Our analysis of *IGF2R* allelic expression in various macaque tissues suggests that preferential monoallelic expression occurs consistently across all tissues except testes, where *IGF2R* expression was wholly biallelic (Fig. 1B).

Like *IGF2R*, *SLC22A2* and *SLC22A3* are also polymorphically imprinted in human term placenta, although their mouse counterparts are imprinted (Ogawa et al. 1993; Xu et al. 1993; Riesewijk et al. 1996; Monk et al. 2006). We observe biallelic expression of macaque *SLC22A2* in placenta and other somatic tissues in all samples analyzed. However, though we do not see polymorphic imprinting, our sample size is small. Macaque *SLC22A3* is polymorphically imprinted, and we anticipate that these genes behave similarly in macaque and human. Intriguingly, macaque *SLC22A1* is imprinted in the placenta and polymorphically imprinted elsewhere, though there is no precedent for this in the human or mouse, where the gene has not previously been shown to be imprinted. Plausibly, *SLC22A1* may also be polymorphically regulated in the human population, albeit restricted to late gestational extraembryonic tissues, which have not previously been examined at this locus.

Given the species variation in imprinting at this cluster, we next considered if an orthologous macaque *AIRN* transcript was present. Though a human homolog of mouse *Airn* has been reported, its in vivo expression and imprinting role is unclear (Oudejans et al. 2001; Yotova et al. 2008). Our attempts to isolate a similar transcript in macaque by 5′/3′ RACE were unsuccessful. However, we have amplified a monoallelic transcript that overlaps *IGF2R* intron 2 and may be indicative of macaque *AIRN* (Supplemental Table 1).

Other maternally imprinted loci examined in the cynomolgus macaque showed expression profiles more closely conserved with their human and mouse counterparts. The *SNRPN* cluster includes genes heavily involved in neural development, as exemplified by cognitive impairments in patients with imprinting defects in this region (Knoll et al. 1989). Like in human and mouse, known paternally expressed genes within the *SNRPN* cluster (*MKRN3*, *MAGEL2*, *NDN* and *SNRPN*) are imprinted in macaque somatic tissues including cerebellum (Nicholls and Knepper 2001), whereas the known maternally expressed counterparts (*UBE3A* and *ATP10A*) are imprinted in a tissue-specific and individual-dependent manner (imprinted *UBE3A* expression: cerebellum only, imprinted expression of *ATP10A*: placenta only) (Fig. 1B).

The product of *PLAGL1*, a paternally expressed imprinted gene, is proposed to be a master regulator of multiple imprinted genes (Varrault et al. 2006) and is imprinted in all tested mouse and human tissues (Arima et al. 2000; Kamiya et al. 2000; Piras et al. 2000). Macaque *PLAGL1* was imprinted in most tissues examined, though imprinted expression in liver shows parental bias (Fig. 1B). In mouse liver, a biallelic alternative transcript of *Plagl1* originates >50 kb upstream of the imprinted *Plagl1* transcript and shares overlapping exons, confounding analysis of *Plagl1* expression in mouse. A similar biallelic transcript, if present in macaques, would potentially contribute to the incomplete imprinting observed (Piras et al. 2000; Valleley et al. 2007).

The *L3MBTL1* locus represents a recently characterized human imprinted gene that is not imprinted in mouse, despite their apparent sequence homology (Li et al. 2005). Imprinting at this locus was acquired after the divergence of primate from rodent, and the status of this gene in macaque helps to further refine this point of divergence. Though *L3MBTL1* imprinted expression is conserved in the macaque, its adjacent genes are generally not imprinted (Fig. 1). *SGK2*, located immediately downstream from *L3MBTL1*, is polymorphically imprinted in macaque, suggesting that it could be imprinted as a bystander to the regulation at *L3MBTL1* (Aziz et al. 2013). Intriguingly, we also noted that imprinted expression of *L3MBTL1* and *SGK2* in adult macaque peripheral blood monocytes also associated with gender, which may warrant future consideration with larger sample sizes (Supplemental Table 1).

A number of imprinted genes in the mouse are thought to originate from a retrotransposition of the X chromosome into an autosomal host gene (McCole and Oakey 2008). Of four such genes (*Nap1l5, Inpp5f* transcript variant 2*, Mcts2*, and *Zrsr1*), three were shown to have corresponding human orthologs (*NAP1L5, INPP5F* transcript variant 2, and *MCTS2*). The fourth, antisense transcript *Zrsr1* in mouse *Commd1* (also known as *Murr1*) arose independently in rodents after a primate-rodent divergence and resulted in *Commd1* imprinting by transcriptional interference (Wang et al. 2004; Zhang et al. 2006; Wood et al. 2007b). With no primate ortholog of *Zrsr1*, it is not surprising that macaque *COMMD1*, as in human, was biallelic in all tissues analyzed (Fig. 1B). At the mouse *Inpp5f* locus, three transcript variants, two of which are imprinted, have been identified (Wood et al. 2007a,b; McCole and Oakey 2008). Conserved in human, imprinted expression of *INPP5F* transcript variant 2 but not *INPP5F* transcript variant 1 (hereafter abbreviated as *INPP5F_v1* and *INPP5F_v2*, respectively) has also been reported in human fetal spinal cord, brain, heart, and tongue (Wood et al. 2007b). Likewise, in macaques, *INPP5F_v1* shows no evidence of imprinted expression, while *INPP5F_*v2 is monoallelically expressed in all extraembryonic and somatic tissues tested (Fig. 1B).

The region from *PEG3-ZNF264* has undergone a number of changes in primates. Compared to its rodent counterpart, the gene distance between *PEG3* and *ZIM2* has become increasingly abbreviated with primate evolution, resulting in the gradual loss of an intervening *Zim1* transcript and the overlap of *ZIM2* and *PEG3* transcripts (Huang and Kim 2009). In macaque placenta, *ZIM2* is polymorphically imprinted, while *ZNF264*, the most distal of these genes, is biallelically expressed in somatic tissues, in agreement with bovine data (Kim et al. 2007). *ZNF264* is imprinted in macaque placenta. It is possible that the increased gene distances between *ZNF264*, *ZIM3*, and the *PEG3* DMR in primates may now include elements that restrict imprinting of *ZNF264* to the extraembryonic lineage (Fig. 1B). While it may be possible that the DMR we have identified may not be the critical control DMR for the *PEG3* cluster, we were unable to find other somatic DMRs in the region (data not shown).

### Conservation of differential methylation at orthologous imprinting control regions (ICRs) in the macaque

**Figure 2.**
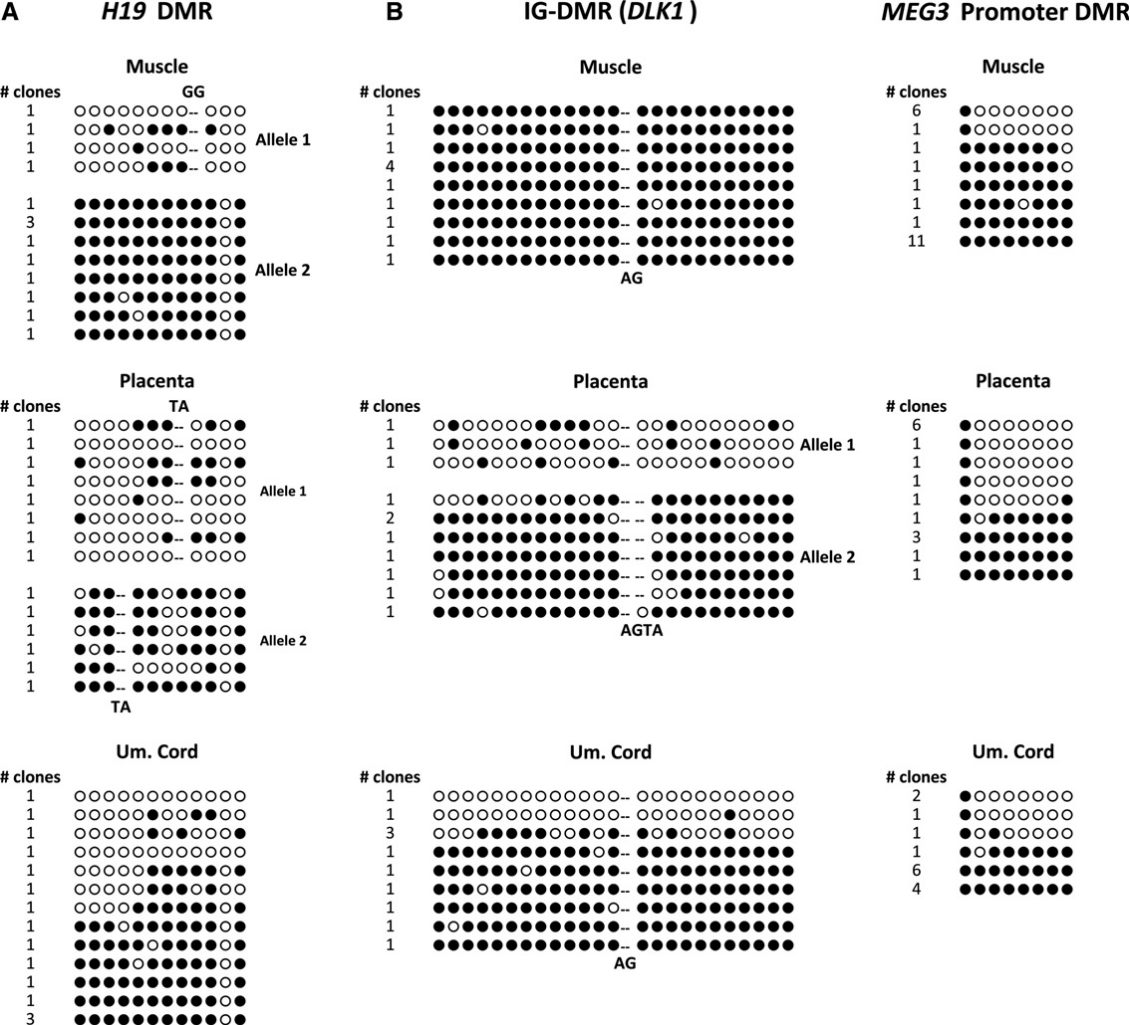
Methylation at paternally imprinted gene clusters. Methylation of DMRs at the *H19* (*A*) and *DLK1* (*B*) clusters in extraembryonic and somatic tissues was obtained by bisulfite sequencing. Although parental/offspring samples were not available, allelic distinction between methylated/unmethylated alleles is consistent between tissues from the same individual and supports imprinting, not random allelic inactivation, at these loci. SNPs around each DMR were used to make the allelic distinctions shown.SNPs around each DMR were used to make the allelic distinctions shown.

**Figure 3.**
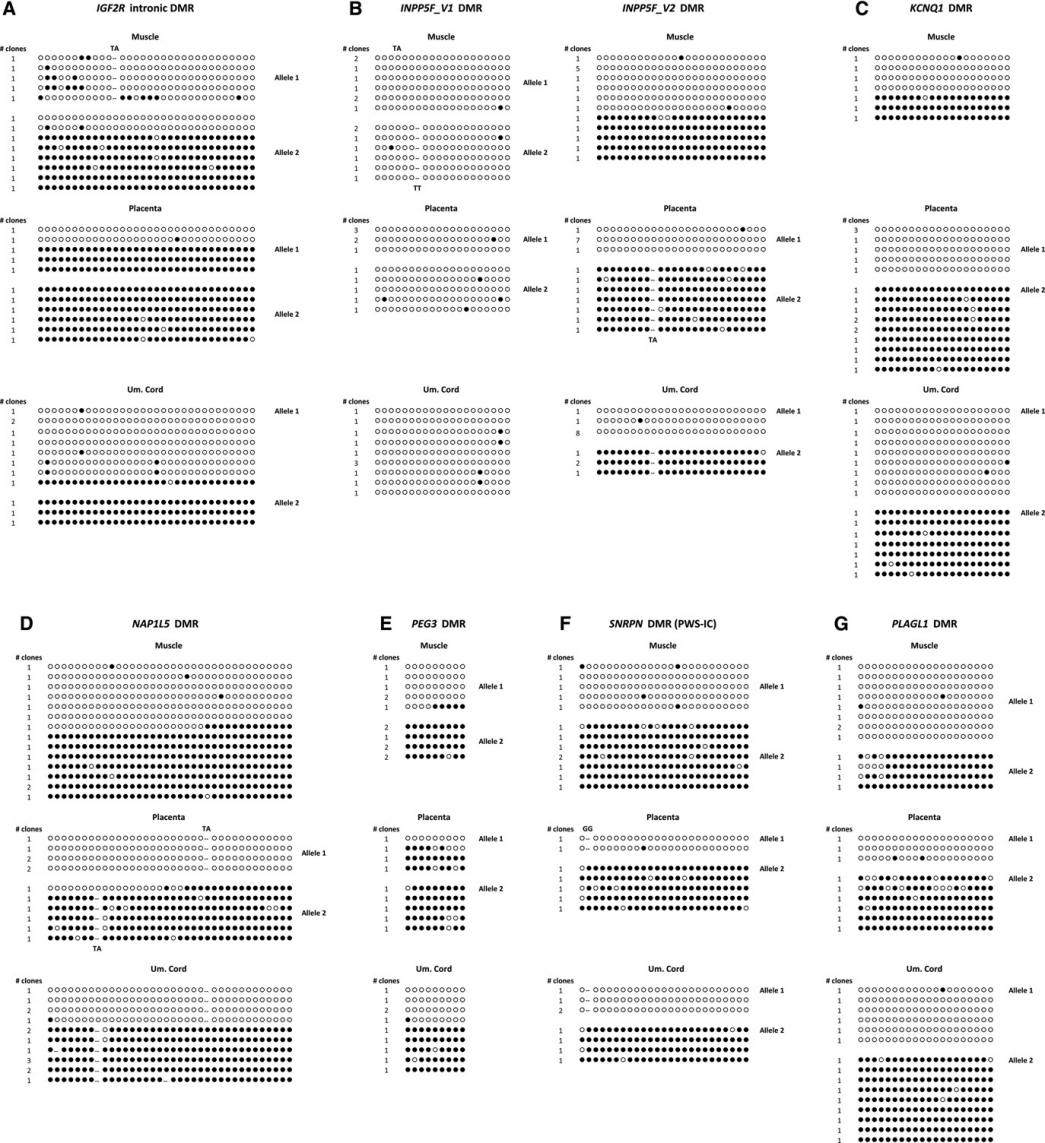
Methylation at known maternally imprinted gene clusters. Differentially methylated ICRs are evident for all maternally imprinted clusters in both somatic and extraembryonic tissues. Individual panels represent DMRs of each cluster as titled. (*A*) *IGF2R* intronic DMR. (*B*) *INPP5F_v1* is biallelically expressed with an unmethylated promoter and is shown alongside the imprinted *INPP5F_v2* for comparison. (*C*) *KCNQ1* DMR. (*D*) *NAP1L5* DMR. (*E*) *PEG3* DMR. (*F*) *SNRPN* DMR (PWS-IC). (*G*) *PLAGL1* DMR. SNPs around each DMR were used to make the allelic distinctions shown.

We next assessed putative ICRs of imprinted gene clusters by bisulfite sequencing and assumed parental origin of methylation at the ICR based on published rodent and human findings (Figs. 2, 3; Supplemental Fig. 5), since no parental macaque samples were available for confirmation. Putative ICR regions were identified using BLAT and VISTA tools against known human and/or mouse ICR regions, with coordinates shown in Supplemental Figure 3 (Kent 2002; Frazer et al. 2004). All genes and DMRs identified in the macaque retained high sequence and syntenic homology, both within individual genes and across the cluster, and were not duplicated elsewhere in the macaque genome.

The macaque *H19* DMR is located ∼2 kb upstream of *H19*, as in human and mouse. Informative cynomolgus samples show distinct differences in the degree of methylation on each parental allele, with the presumed paternal allele more heavily methylated, although sporadic CpGs interspersed within the putative DMR show an opposite pattern within each clone (Fig. 2A). Fujimoto et al. (2005) previously showed that the *H19* DMR in juvenile rhesus muscle showed variable methylation, although this was determined by methylation-specific PCR and did not show base resolution methylation (Fujimoto et al. 2005; Mitalipov et al. 2007). In human, base resolution bisulfite sequencing of the *H19* DMR in lymphocytes also showed variable methylation between clones, although the averaged methylation across the region approximates 50% (Kerjean et al. 2000; Borghol et al. 2006). Our results demonstrate that this complexity at the human *H19* DMR is also seen in the macaque.

The *DLK1* IG-DMR and *MEG3* DMR were located by homology to their known human DMRs (Kagami et al. 2008, 2010). In the human, long-range interactions between these DMRs may establish the *MEG3* DMR as a secondary ICR in somatic tissues, while the IG-DMR alone is responsible for placental imprinting (Kagami et al. 2010). Consistent with a more prominent role for the *MEG3* DMR in somatic tissues, the macaque IG-DMR is differentially methylated in term placenta and umbilical cord; yet in adult somatic tissue (Fig. 2B; Supplemental Fig. 5), this region no longer retains allele-specific methylation. This suggests a diminished role for IG-DMR maintenance in the adult macaque following establishment of the *MEG3* DMR, which is differentially methylated in extraembryonic and analyzed somatic tissues. This differs markedly from the human IG-DMR, where differential methylation appears to be retained in adult somatic tissues (Kagami et al. 2008, 2010), although hypermethylation has been observed in the human placenta (Murphy et al. 2012). In mouse, the more distal section of the IG-DMR gradually loses complete parental distinction of methylation, with somatic tissues of mid-gestation embryos showing a marked increase in maternal methylation (Sato et al. 2011). Interestingly, there is little sequence conservation between primates and rodents at the respective established IG-DMRs, with homology largely limited to the 3′ end of the DMR (Paulsen et al. 2001). Comparisons of the homologous macaque and human IG-DMR sequences also suggest that despite high overall sequence conservation of >96%, CpG site homology is far lower at ∼50% (data not shown), suggesting that additional mechanisms and sequence features may be required to maintain differential methylation at the IG-DMR and *MEG3* DMR in each species.

The *SNRPN* DMR is known to be maternally methylated in juvenile rhesus macaque muscle and ES cell lines (Fujimoto et al. 2005; Mitalipov et al. 2007). Our results in cynomolgus macaque tissues are in agreement, and we further demonstrate that differential methylation is also seen in placenta and umbilical cord (Fig. 3). We also show that the ICRs of *KCNQ1* and *IGF2R* are differentially methylated in somatic tissues, although some hypermethylation is evident in placenta at the *IGF2R* DMR, perhaps reflective of a reduced requirement for DMR maintenance in the extraembryonic lineage. This was also observed at the *PEG3* DMR (Fig. 3; Supplemental Fig. 5). Imprinted genes *PLAGL1*, *NAP1L5*, and *INPP5F_v2* were also associated with differential methylation at their promoter regions (Fig. 3). In comparison, the nonimprinted *INPP5F_v1* promoter was unmethylated, even though this variant shares exons with the imprinted *INPP5F_v2* gene.

### Determination of germline imprinting in the primate

Germline imprints are DMRs acquired during gametogenesis (Ferguson-Smith 2011) and depend on DNA methyltransferases to establish and maintain these modifications (Tucker et al. 1996; Kaneda et al. 2004b; Weaver et al. 2009). *DNMT* mutant mice exhibit loss of methylation and perturbed imprinting (Biniszkiewicz et al. 2002; Kaneda et al. 2004a; Kato et al. 2007), and mice knockouts for specific germline DMRs have demonstrated that these serve as imprinting control regions for the locus (Thorvaldsen et al. 1998; Bielinska et al. 2000; Fitzpatrick et al. 2002; Lopes et al. 2003; da Rocha et al. 2008). The mouse has been the primary species for determining DMR germline origin (Supplemental Table 6). A small number of human germline DMR analyses have been assessed at the *DLK1*, *H19*, *KCNQ1*, and *SNRPN* loci at orthologs to the murine DMRs, though this may be subject to inherent methylation defects within the accessible patient samples (Geuns et al. 2003, 2007a,b). More recently, whole-genome bisulfite sequencing of human oocytes suggested hypermethylation across all known maternally derived ICRs (Okae et al. 2014), and we sought to establish if there were common features of these regions between human and nonhuman primates.

**Figure 4.**
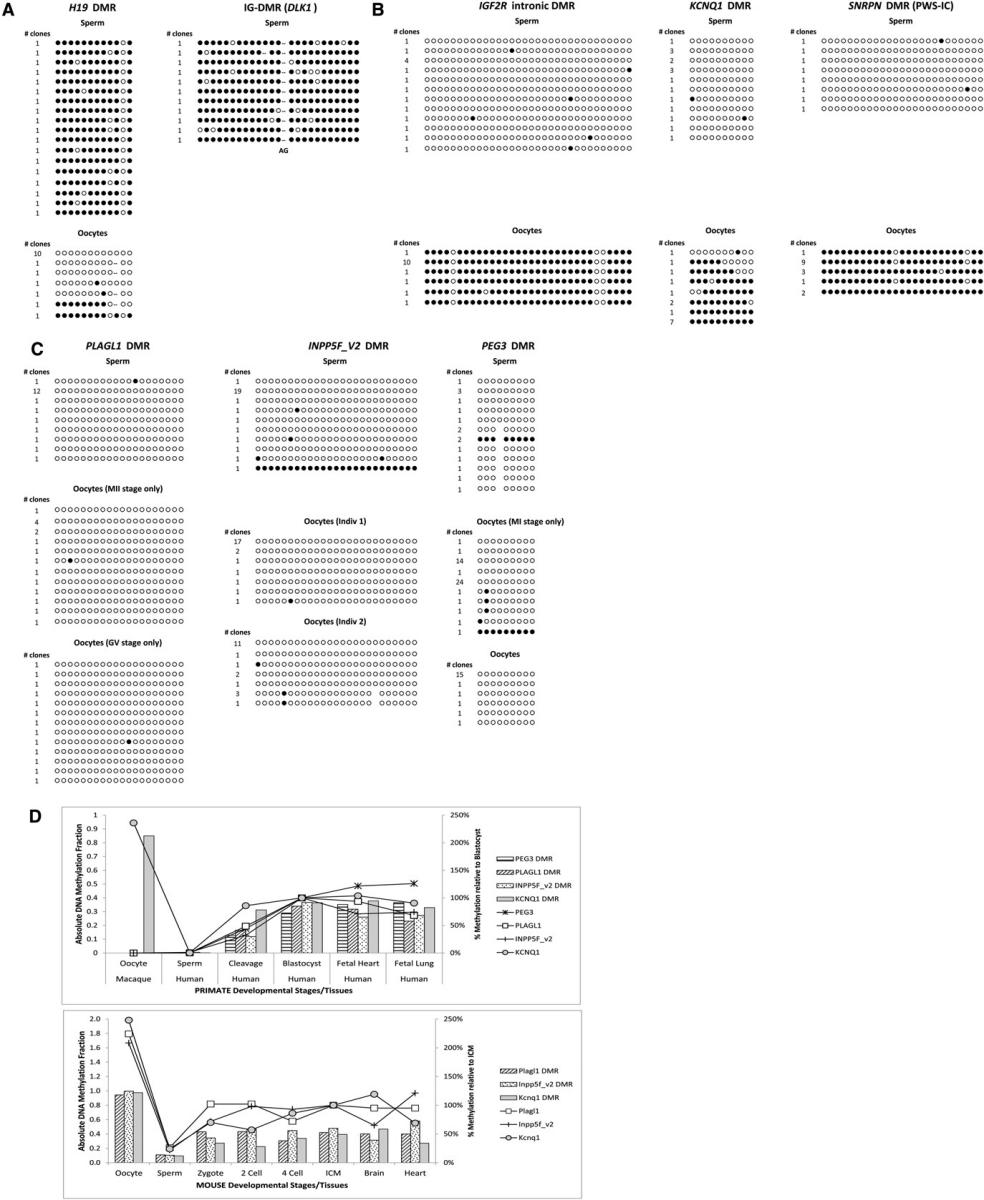
Germline imprints in the cynomolgus macaque. DNA methylation in macaque gametes fall into three distinct groups of paternally (*A*) and maternally (*B*) acquired germline imprints, and a third group (*C*) with delayed maternal imprint acquisition. Except for *PLAGL1* (*lower* oocyte image: GV only; *upper* oocyte image: MII only), all other oocyte samples comprised of pooled stages (GV, MI, and MII). In *D*, macaque oocyte methylation is shown alongside meta-analysis of data from Smith et al. (2014), showing that the lack of oocyte methylation at *PLAGL1, PEG3*, and *INPP5F_v2* (patterned bars) is consistent with human, where methylation is gradually acquired across the primate embryonic cleavages, and only completed by the blastocyst stage. In contrast, mouse homologs show that imprinted methylation is present in oocytes and already complete in the early zygote. The intronic *KCNQ1*/*Kcnq1* DMR (gray bar) is shown for comparison. No data were available for mouse *Peg3*. (*Left* axis, bar chart) Absolute DNA methylation, (*right* axis, line graph) percent DNA methylation relative to level in ICM/blastocyst.

Having analyzed macaque tissues for differential methylation at putative DMRs, we next used primate gametes to assess the germline origin of these DMRs. Previously, we determined that the primate-specific imprinted gene, *L3MBTL1*, associates with a maternal germline imprint (Aziz et al. 2013). In this study, all eight DMRs showed germline methylation consistent with the expected parental origin of the locus (Fig. 4A–C). We also analyzed macaque oocytes and found reciprocal methylation at four of the eight loci examined, in line with their expected germline origin. Insufficient oocyte material was available to assess the *DLK1* IG-DMR. Oocytes were collected from ovarian stimulated and normal cycling females, cleaned of cumulus cells and debris following enzymatic incubation and mechanical pipetting, then pooled by individual for subsequent analysis. In assurance of appropriate technical care, oocytes were largely unmethylated at the paternally imprinted *H19* DMR, consistent with the human germline DMR status (Borghol et al. 2006).

Similarly, at *SNRPN*, *IGF2R*, and *KCNQ1*, known maternal germline inherited DMRs in rodents were also fully methylated in macaque oocytes (Fig. 4B). At the human *SNRPN* locus, the timing of maternal imprint acquisition is debated and centers on the presence or absence of a complete imprint by the meiosis II (MII) stage in oocytes (El-Maarri et al. 2001; Geuns et al. 2003). Our sample at SNRPN comprised three oocyte stages (GV, MI, MII) and was fully methylated, in agreement with the analysis of Geuns et al. (2003) on human oocytes.

Intriguingly, we expected maternal germline methylation at three other loci (*PLAGL1*, *PEG3*, and *INPP5F_v2*), but instead found these largely unmethylated. To confirm that this was not due to sampling limitations, we repeated this on an independent sample, with similar outcomes (Fig. 4C). The lack of methylation in macaque oocytes at these loci suggests that the imprinting mechanisms conferring DMR identity at a germline stage may not be fully conserved between species. In the mouse, DMRs of *Plagl1*, *Peg3*, and *Inpp5f_v2* are maternally inherited in the germline (Supplemental Table 6). Concordant with this expectation, macaque sperm at these DMRs are unmethylated (Fig. 4C). Yet in macaque oocytes, we found no evidence of maternal methylation, indicating that this is acquired post-fertilization, since allele-specific differential methylation was seen in somatic tissues (Fig. 3). Recently available data on the methylomes of human gametes and early embryos suggests that there are subtle but distinct differences between human and rodent development, with human genome-wide remethylation complete only in the post-implantation embryo (Guo et al. 2014; Smith et al. 2014). Meta-analysis of data from Smith et al. (2014) indicated that the three imprinted loci which, in macaque, were unmethylated in the female germline, acquired methylation during the early cleavage to blastocyst stages in human (Fig. 4D). In contrast, the intronic DMR at *KCNQ1,* which we show is germline in the macaque, maintains methylation from the cleavage stages through to later development. These findings are consistent with the lack of methylation we observe in the macaque oocyte at *PLAGL1*, *PEG3*, and *INPP5F_v2* and suggest that maternal-specific methylation may indeed be acquired during these early primate post-fertilization divisions. No overlapping DMR coverage was found in the oocyte data sets from Guo et al. (2014). It remains to be determined if alternative maternal-specific marks, such as histone modifications, may already be in place in gametes.

## Discussion

From a broad survey of known human/mouse imprinted genes in the nonhuman primate, we have found that at most clusters, imprinted expression and differential methylation is concordant with both the widespread and tissue-specific patterns observed in human and mouse. Our analysis also revealed previously unrecorded tissue-specific imprinting in healthy adult primate tissues such as kidney, muscle, pancreas, and testes.

This study also uncovered novel primate-specific imprinting features:

At the expression level, a novel transcript of *CDKN1C* appears to be specific to the cynomolgus macaque. The *CDKN1C* transcript variant results from alternative splicing between exon 1 and 2, though the expected splice junction retains the classic GT-AC donor-acceptor sequence (data not shown). In the human genome, its corresponding sequence is characterized by a highly repetitive region. Between primates, segmental duplications are associated with repetitive sequences, regions of high instability, and recombination proposed to have contributed to primate evolution (Kehrer-Sawatzki and Cooper 2008). Such alterations may promote transcriptional or regulatory changes. Furthermore, macaque-human synteny maps localize the *CDKN1C*-containing *KCNQ1* cluster <500 kb from a chromosomal inversion in macaques, consistent with a possible shared role of direct repeats as well as longer range enhancer elements in directing transcriptional splicing (Ventura et al. 2007).

Polymorphic imprinting has been described for IGF2R in humans. At this cluster, we demonstrate that macaque imprinting appears to have features that are intermediate between human and mouse. It is clear that the *IGF2R* intron 2 DMR found in human is also present in the macaque. However, the polymorphic imprinting of *IGF2R* that has been described for human was not evident in the macaque tissues analyzed in our study. It therefore appears that imprinted *IGF2R* is evident in at least this old world primate species (Fig. 1; Supplemental Table 1). The species-specific difference may be genetically conferred; however, we note that a single ZFP57 binding site, required for the maintenance of imprints in early development in rodents (Li et al. 2008), is shared between the human and macaque in intron 2 of *IGF2R*, hence this is unlikely to be the cause of the species-specific difference.

At the *IGF2R* locus, a syntenic disruption occurs between human and macaque. Macaque *IGF2R* is located more proximal to a neocentromere feature than its human equivalent—this association might partially account for the differences observed in this cluster (Ventura et al. 2007; Rocchi et al. 2009). Further comparative genomic mapping may highlight features that contribute to imprinting regulation control.

We also observed some potential differences between human and macaque in the retention of the DMR status at the IG-DMR in somatic tissues, suggesting that the more conserved methylation status of the nearby *MEG3* DMR might function as the primary ICR in somatic tissues.

In addition, we analyzed *L3MBTL1* and its downstream neighbors for evidence of imprinting. Our macaque data show conserved *L3MBTL1* imprinting between primate species and also expand the present understanding of tissue-specific imprinting at this locus, which is not imprinted in mouse or marsupial (Aziz et al. 2013).

Our understanding of genomic imprinting mechanisms is based primarily on genetic and epigenetic studies in mouse, with conservation of all DMRs found between mouse and human somatic tissues. Analysis of patients with parental origin effects such as Beckwith-Wiedemann and Prader-Willi/Angelman syndromes indicates that these murine germline-derived DMRs are also functionally conserved ICRs in human. However, the developmental origins of primate ICRs are not well established, and we were keen to verify if previously ascertained or assumed human germline DMRs were indeed germline in origin.

The limited availability of human gametes has precluded analysis from all but two maternal inherited loci DMRs (*KCNQ1*, *SNRPN*) and two paternally inherited loci (*DLK1*, *H19*) (Kerjean et al. 2000; El-Maarri et al. 2001; Geuns et al. 2003; Borghol et al. 2006). While reduced representative bisulfite sequencing (RRBS) data on human gametes and early embryos have recently become available, these techniques do not guarantee coverage at all loci of interest but have provided a global perspective on DNA methylation at each developmental stage (Guo et al. 2014; Smith et al. 2014). Whole-genome bisulfite sequencing of human oocytes suggests that maternally derived ICRs are hypermethylated in the human (Okae et al. 2014), including the orthologs of the three loci that we show with delayed maternal imprinting in macaque oocytes. Although averaged rather than specific locus detail is presented, this may point to species-specific differences in the acquisition of imprints between humans and nonhuman primates.

We obtained oocytes from both normal and ovarian stimulated female macaques and manually removed surrounding cumulus cells by multiple washes and pipetting with increasingly narrower bores. The presence of two clones with partial methylation at the *H19* DMR in oocytes suggests the presence of maternal germline epimutation, or that despite our best efforts, our oocyte sample may have rare somatic cell contamination (no contamination was evident at other loci using the same 46-cell sample). Nonetheless, >87% of the reads represented unmethylated clones, and we interpret this to mean that, as in the mouse, the maternal allele at the *H19* DMR is predominantly unmethylated in the female germline.

In mice, the exact acquisition timing of maternal germline imprints differs between loci but is completed by the MII stage (Obata and Kono 2002; Hiura et al. 2006). While superovulation in mice can also contribute to a dose-dependent effect on imprinting, this is evident only with oocytes derived from overstimulation, with the *Snrpn*, *Peg3*, and *Kcnq1* DMRs showing loss of methylation in oocytes derived from high dose ovulation protocols (Market-Velker et al. 2009). In macaque, these potentially sensitive loci appear unaffected by our stimulation protocol, since the majority of imprinted loci examined show the expected methylation patterns. At *SNRPN* and *KCNQ1*, normal maternal imprints were evident in superovulated macaque oocytes. Results for DMRs at *H19, KCNQ1, IGF2R*, and *SNRPN* also confirmed canonical germline imprinting, in agreement with known imprints from nonprimate species.

Unexpectedly, three DMRs of established germline origin in rodents did not acquire their DMR status in the macaque germline. At the *PLAGL1*, *INPP5F_v2*, and *PEG3* DMRs, no methylation was found in either the sperm or oocytes examined. These findings question the central dogma that DNA methylation at all ICRs is established during gametogenesis and is subsequently resistant to the genome-wide demethylation events that occur post-fertilization. While genomic rearrangements between species, particularly at the *PEG3* cluster, may not conclusively point toward the regions analyzed as the sole or critical DMR for the cluster, we did not find other somatic DMRs at the *USP29* nor *ZNF264* promoters within the cluster, suggesting we have not missed other regions with the potential to be germline, unless they are transitory.

Additionally, we do not anticipate that the germline absence of methylation observed at these loci is an artifact associated with oocyte maturation or superovulation, since *PLAGL1* methylation was absent in an exclusively MII oocyte sample from one individual, yet *SNRPN* methylation was complete in a separate oocyte sample that included less mature GV oocytes. Neither do we suspect conversion failure at rare non-CG methylated primer targets. Instead, we postulate that post-oocyte acquisition of methylation at *PLAGL1*, *INPP5F_v2*, and *PEG3* is a primate phenomenon. It is notable that in the human methylome, global DNA hypomethylation was seen in the early human cleavage stages (Guo et al. 2014; Smith et al. 2014). More specifically, we looked at imprinted genes with coverage in these methylome data sets. Consistent with our findings, DNA methylation at *PLAGL1*, *PEG3*, and *INPP5F_v2* was only partially acquired by the cleavage stage, whereas the *KCNQ1* DMR was methylated at levels similar to somatic tissues by this early developmental time point and corresponding full maternal imprints already present in oocytes (Smith et al. 2014).

When compared to other imprinted loci with parental-specific DNA methylation already acquired in the germline, it is possible that parental histone marks may be different at *PLAGL1*, *INPP5F_v2*, and *PEG3*, leading to the post-fertilization differential recruitment of DNMTs. Notably, the DNMT profile in primates differs from mouse, with a marked relative reduction of DNMT3 de novo methylases in primates at the oocyte stage, during which imprints are established in rodents (Huntriss et al. 2004; Vassena et al. 2005). However, within this study, we were unable to assess allele-specific histone modifications. In primates, DNMT3A levels rise ∼10-fold as oocytes progress to fertilized pronuclei-stage zygotes. Plausibly, this surge of DNMT enzymes may assist in completion of germline DMR methylation while the parental chromosomes are still separated (Vassena et al. 2005). Alternatively, there may be species-specific genetic differences in the occurrence of oocyte-specific upstream promoters, thought to drive transcription required for oocyte-specific acquisition of methylation at imprinted ICRs (Smallwood and Kelsey 2012).

What might be the implications of deferred maternal methylation at these genes in primates? Prior to the maternal-zygotic transition, the emphasis of regulatory mechanisms depends more on post-transcriptional activities, not zygotic transcription. In primates, the zygotic genome is activated later than the very early two-cell activation found in mice. To this end, we speculate that the selection for pre-fertilization imprint acquisition via maternal germline methylation is relaxed in the primate, with little negative impact on the early embryo. It is noteworthy that the early germline-derived imprinted X inactivation described in mice is not found in human (Okamoto et al. 2011). The dosage of imprinted genes such as *PLAGL1* and *PEG3* is important for postnatal metabolism. Maternal modulation of their epigenetic status after, rather than before fertilization, may be permissible in species such as primates with a later onset of zygotic transcription. This might provide an adaptive programming mechanism sensitive to environmental resources that provides a selective advantage to offspring.

With our findings, it is evident that though imprinted expression is largely conserved between Eutherians, the timing and exact mechanisms employed might involve subtle yet profound differences between species. We suggest that these findings in a nonhuman primate emphasize the importance of post-fertilization events in imprinting control (Hanna and Kelsey 2014).

## Methods

### Nonhuman primate use

Adult cynomolgus macaques were housed at the A*STAR Non-human Primate Facility. Animals 5–10 yr of age and surplus to the breeding colony requirements at the center were euthanized, and tissues collected for further analysis. Cynomolgus birth tissues were collected from a breeding facility in Vietnam. All animal protocols and experiments were approved and conducted in accordance with requirements of the SingHealth Institutional Animal Care and Use Committee (IACUC #2009/SHS/509). No primate parent-offspring pairs were available for parent-specific allelic expression analysis.

### Ovarian stimulation and oocyte retrieval

Regular monthly cycling female macaques (*n* = 6) between 5 and 8 yr of age were administered with follicle stimulating hormone (rhFSH, 5.5–7 units/kg, twice daily, GONAL-f, Merck-Serono) for 7–10 d from the onset of menstruation. From Day 7, luteinizing hormone (rhLH, 20 units/kg, twice daily, Luveris, Merck-Serono) and gonadotropin releasing hormone antagonist (GnRH antagonist, 5 μg/kg, once daily, Cetrotide, Merck-Serono) were also delivered intra-muscularly. Animals were monitored by ultrasound from Day 8 and administered with a single chorionic gonadotropin-α dose when more than five follicles (>3 mm) were visible (rhCG-a, 1000 units, Ovidrel, Merck-Serono). Twenty-four to twenty-seven hours later, animals were subjected to an ovariectomy. Cumulus-oocyte complexes (COCs) were aspirated from follicles and placed in equilibrated KSOM medium (Sigma-Aldrich) overnight at 37°C, 5% CO_2_ to allow for maturation of any immature oocytes. The following morning, healthy COCs were placed in M2 medium (Sigma) containing 0.025% trypsin and 1 mg/mL hyaluronidase, and cumulus cells were removed by mechanical pipetting. Denuded oocytes were washed in consecutive rounds of M2 medium and classified by nuclear maturity. Oocytes from the same individual were pooled for DNA analysis, numbers as follows: *H19*, *INPP5F* (Indiv. 1), and *SNRPN*, *n* = 46 (GV-1, MI-25, MII-20); *INPP5F* (Indiv. 2), *n* = 17; *PEG3*, *n* = 14; *PLAGL1*, GV-6, MII-12; *KCNQ1*, *n* = 36 (MI-28, MII-8).

### Sperm retrieval

Testes were retrieved from adult male macaques euthanized for nonfertility-related studies at the animal facility. Surface blood vessels were bled to reduce blood contamination, washed, and then placed in a dish with fresh PBS. To release sperm, the epididymis was cut repeatedly with scissors and gently agitated for 10 min at room temperature to allow motile sperm to swim out. This PBS suspension was then spun down at 1500*g*, 15 min, 4°C to remove excess volume and the pellet used for DNA analysis.

### DNA isolation, bisulfite conversion, and PCR

DNA isolated from sperm was based on a two-step lysis procedure as previously described (Alcivar et al. 1989; Carracedo 2005). Contaminating somatic cells were first lysed by placing the sperm pellet in buffer containing 0.8 mg/mL Proteinase K (Roche Applied Sciences), 30 min at 37°C. Subsequently, sperm were lysed by addition of fresh buffer with 0.8 mg/mL Proteinase K and 40 mM DTT, incubated overnight at 55°C, and used for standard DNA extraction. Oocytes were subject to multiple freeze-thaw cycles, then digested for 90 min in buffer containing 0.25 mg/mL Proteinase K and 2.5 μM SDS at 37°C—protocol modified from Zuccotti and Monk (1995). Proteinase K was inactivated at 98°C for 15 min. The lysed cell extract was used directly for bisulfite conversion. Prior to DNA extraction, whole-blood samples were subject to lysis of red blood cells in a hypotonic buffer (20 mM Tris-Cl, pH 8.0, 0.1 M NaCl, 25 mM EDTA, 0.5% SDS). The white cell pellet was lysed with proteinase K and used for standard DNA extraction. All other solid tissues were first homogenized in gentleMACS M tubes (Miltenyi Biotec) before standard DNA extraction. Bisulfite conversions were done according to manufacturers’ protocols (Zymo EZ DNA Methylation Gold or Qiagen EpiTect Bisulfite Kit). The SequalPrep Long Polymerase PCR kit (Invitrogen) was used for all bisulfite-PCRs, using the recommended PCR program and 40 cycles. Picked clones for each sample were capillary-sequenced and analyzed using a BiQ Analyzer (Bock et al. 2005). Bisulfite conversion efficiencies for all samples considered ranged from 95% to 100%, with an overall average of 99%, comparable to the reported efficiencies of the bisulfite conversion kits. Capillary sequences for a single sample were mapped to the in silico bisulfite converted reference genome, and conversion efficiencies were calculated by counting the number of unconverted, non-CpG cytosine residues in the amplicon, against the total number of non-CpG cytosines in the original unconverted sequence. Occurrences of these nonconverted sequences were rare, and we note that they cannot formally be distinguished from C/T polymorphisms that may exist on the parental alleles. Animals used are not inbred, and any allelic differences shown on the bisulfite lollipop diagrams were determined at a non-C/T SNP present within the amplicon used for bisulfite sequencing. If any, identical capillary sequences of separate clones were taken to be nonindependent clones resulting from PCR amplification—only a single representation of these clones is shown in each bisulfite lollipop diagram, with the number of clones clearly shown by the side.

### SNP analysis

To identify informative individuals for subsequent analysis of allele-specific expression, genomic DNA surrounding exon regions were amplified by PCR and sequenced. Novel SNPs were identified by alignment, using the rhesus macaque genomic sequence as reference (Rhesus Genome, version rheMac2). These SNPs are available through NCBI dbSNP (accession numbers in Supplemental Table 10).

### RNA isolation, reverse transcription and pyrosequencing for allele-specific expression

Tissues for RNA extraction were homogenized in TRIzol (Invitrogen) and processed according to the manufacturer's protocol. Following phase separation, the aqueous phase was further treated with DNase I and purified on RNeasy mini columns according to the manufacturer's protocol (Qiagen). Reverse transcription was primed with random hexamers (Hi Capacity cDNA Reverse Transcription Kit, Applied Biosystems) and cDNA used for subsequent PCR with AccuPrime Taq (Invitrogen) for 30–35 cycles. RT-PCR products were subjected to standard capillary sequencing and the allele specificity of expression determined by the presence or absence of multiple nucleotide peaks at a predetermined polymorphic location. Pyrosequencing primers were designed to cover the same polymorphism but included a 5′ biotin primer on one end for capture on sepharose beads. Briefly, cDNA was amplified with HotStarTaq Master Mix (Qiagen) at 55°C for 40 cycles. The RT-PCR product was then bound to sepharose beads via the biotin tag and denatured to generate single-stranded DNA to allow annealing of an internal sequencing primer. Pyrosequencing was performed in AQ mode on a Qiagen PyroMark Q24 machine using 10 μL of amplified cDNA product and 0.3 μM of sequencing primer.

## Data access

*M. fascicularis* SNPs used in this study have been uploaded to NCBI dbSNP (www.ncbi.nlm.nih.gov/SNP), with reference to assembly GCF_000364345.1 (Macaca_fascicularis_5.0), using the following accession numbers: ss1414417769, ss1414417873, ss1536213772. These SNPs are also reported in Supplemental Table 10. Cynomolgus-specific *CDKN1C* transcripts from adult tissues have been submitted to NCBI GenBank (www.ncbi.nlm.nih.gov/genbank/) with accession numbers KP238484 (liver), KP238485 (muscle), KP238486 (kidney), KP238487 (lung). Capillary sequence data used in this study are accessible through NCBI Trace Archive (www.ncbi.nlm.nih.gov/Traces) under the TI accession numbers 2340903719–2340904081. Qiagen PyroMark data is available online in the Supplemental Material.

## Supplementary Material

Supplemental Material
