# Supplementary material for: Germline and somatic imprinting in the nonhuman primate highlights species differences in oocyte methylation
Source: Genome Res. 2015 May;25(5):611–23. doi: 10.1101/gr.183301.114 (PMC4417110; doi:10.1101/gr.183301.114)
Supplement: Supplemental Material [file supp_25_5_611__index.html]

Germline and somatic imprinting in the nonhuman primate highlights species differences in oocyte methylation — Germline and somatic imprinting in the nonhuman primate highlights species differences in oocyte methylation — Supplemental Material 

# Germline and somatic imprinting in the nonhuman primate highlights species differences in oocyte methylation

## Supplemental Material

**Files in this Data Supplement:**

- Supplemental Data.pdf
- Supplemental Information.docx
- Supplemental Material.docx
