# Supplementary material for: Germline and somatic imprinting in the nonhuman primate highlights species differences in oocyte methylation
Source: Genome Res. 2015 May;25(5):611–23. doi: 10.1101/gr.183301.114 (PMC4417110; doi:10.1101/gr.183301.114)

Supplementary File 2
Allele-Specific Expression - Pyrosequencing Data

Germline and Somatic Imprinting in the Non-Human Primate Uncovers Primate-Specific Acquisition

Clara Y. Cheong[1], Keefe Chng[1, •], Shilen Ng[1,•], Siew Boom Chew[1, •], Louiza Chan[1], Anne C Ferguson-Smith[1,2]

[1]Growth, Development and Metabolism Program, Singapore Institute for Clinical Sciences, Agency for Science, Technology and Research (A-STAR), 30 Medical Drive, Singapore 117609
[2] Department of Genetics, University of Cambridge, Downing Street, Cambridge CB2 3EH, UK

• Present affiliations; KC: Crown Bioscience Inc., 3375 Scott Blvd., Suite 108, Santa Clara, CA 95054 ; SN: Health Sciences Authority, 3 Biopolis Drive, Synapse, Singapore 138623; SBC: Syngenta APAC Pte Ltd, 50 Science Park Road, #06-01, The Kendall, Singapore 117406

PLAGL1
Allele Specific Expression - cDNA
Qiagen Pyrosequencing

PLAGL1: Liver

Well: B1
Assay: Rhesus_PLAGL1-V2
Sample ID: F6_L
Sequence to analyze: TTAAKCTGTCTGGGGCAGCAGCAGCAAGAA

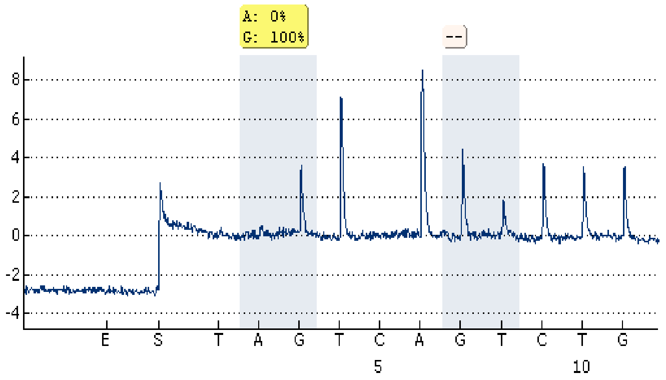

Well: B2
Assay: Rhesus_PLAGL1-V2
Sample ID: M2_L
Sequence to analyze: RTTAAKCTGTCTGGGGCAGCAGCAGCAAGAA

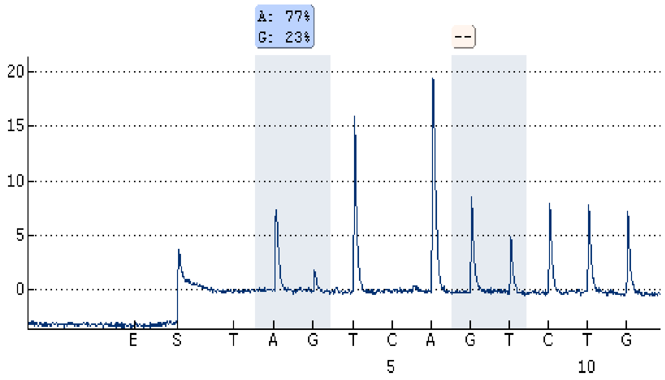

PLAGL1: Kidney

Well: B3
Assay: Rhesus_PLAGL1-V2
Sample ID: F4_K
Sequence to analyze: RTTAAKCTGTCTGGGGCAGCAGCAGCAAGAA

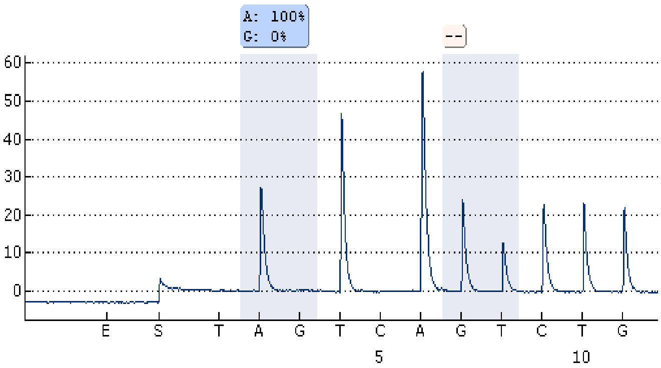

Well: B4
Assay: Rhesus_PLAGL1-V2
Sample ID: F6_K
Sequence to analyze: RTTAAKCTGTCTGGGGCAGCAGCAGCAAGAA

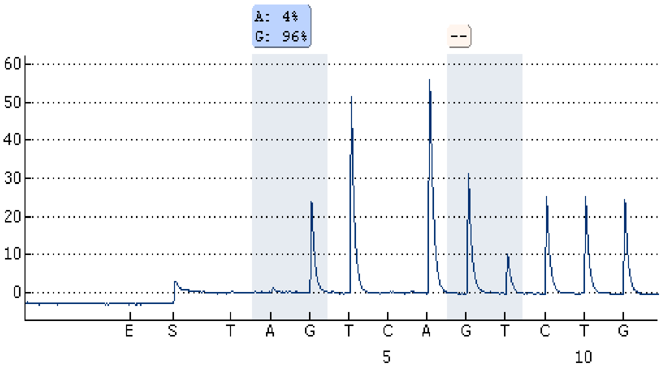

Well: B5
Assay: Rhesus_PLAGL1-V2
Sample ID: M2_K
Sequence to analyze: RTTAAKCTGTCTGGGGCAGCAGCAGCAAGAA

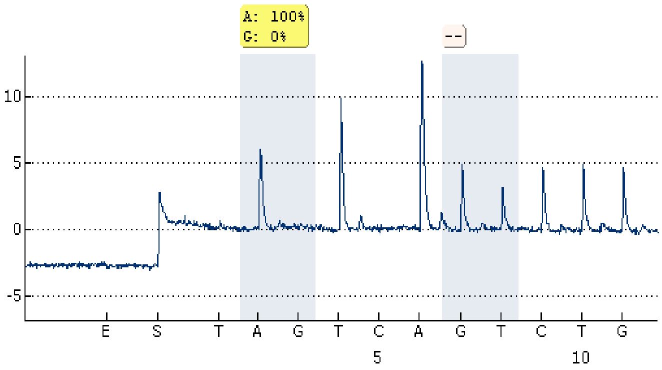

PLAGL1: Pancreas

Well: B6
Assay: Rhesus_PLAGL1-V2
Sample ID: F4_P
Sequence to analyze:
RTTAAKCTGTCTGGGGCAGCAGCAGCAAGAA

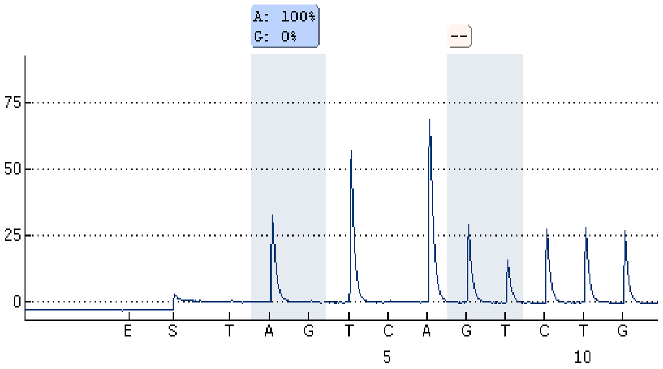

Well: B7
Assay: Rhesus_PLAGL1-V2
Sample ID: F6_P
Sequence to analyze: RTTAAKCTGTCTGGGGCAGCAGCAGCAAGAA

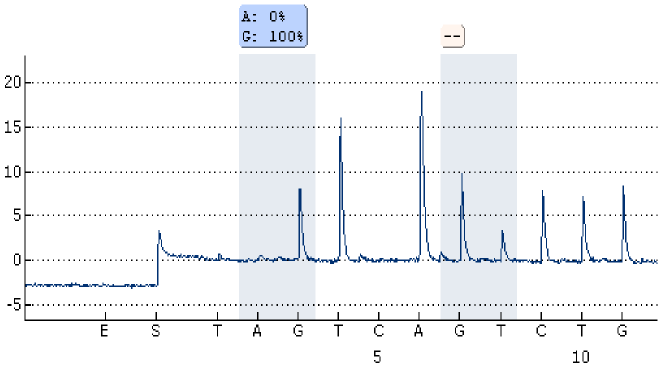

Well: B8
Assay: Rhesus_PLAGL1-V2
Sample ID: M2_P
Sequence to analyze: RTTAAKCTGTCTGGGGCAGCAGCAGCAAGAA

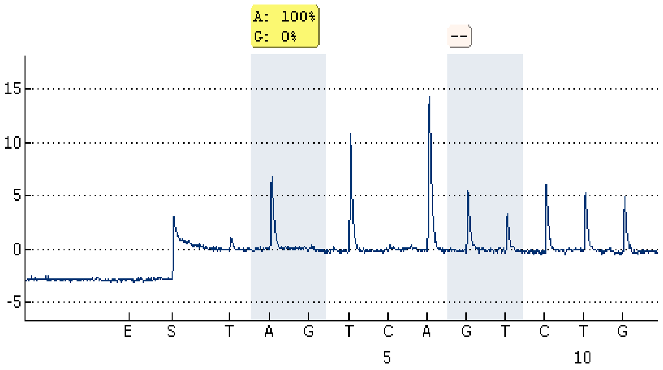

IGF2
Allele Specific Expression - cDNA
Qiagen Pyrosequencing

IGF2: Liver

Well: C8
Assay: IGF2
Sample ID: M1_L
Sequence to analyze: WGTCACAATTGGAGAACCCACATTGG

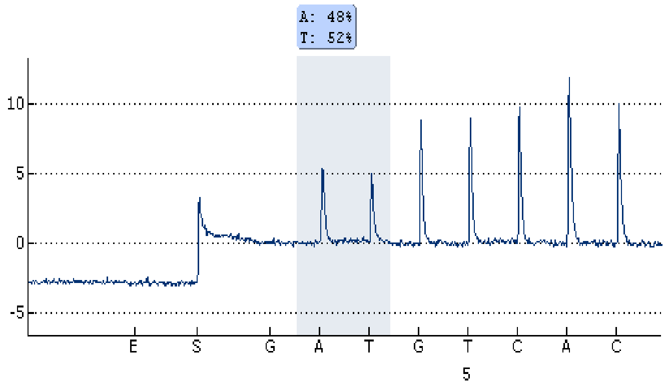

Well: C9
Assay: IGF2
Sample ID: M2_L
Sequence to analyze: WGTCACAATTGGAGAACCCACATTGG

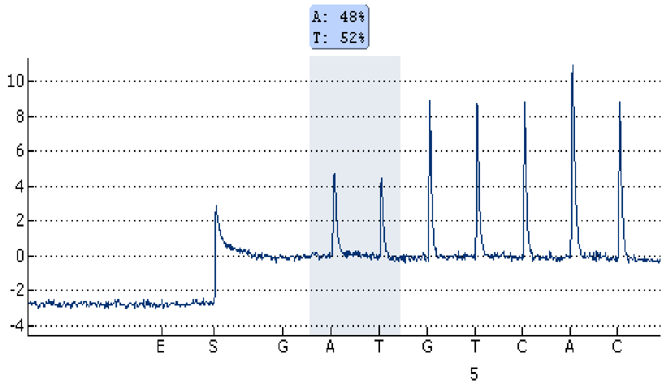

IGF2: Kidney

Well: C12
Assay: IGF2
Sample ID: M1_K
Sequence to analyze: WGTCACAATTGGAGAACCCACATTGG

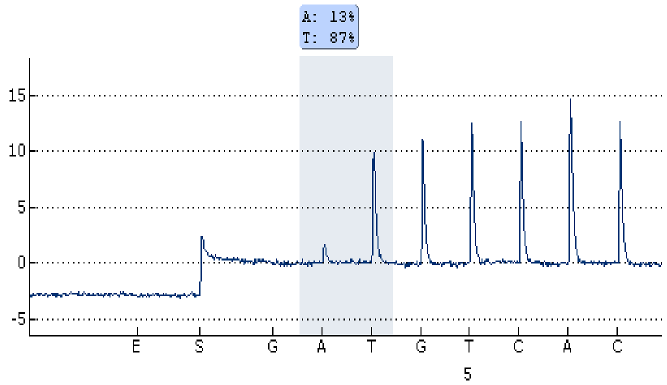

Well: D1
Assay: IGF2
Sample ID: M2_K
Sequence to analyze: WGTCACAATTGGAGAACCCACATTGG

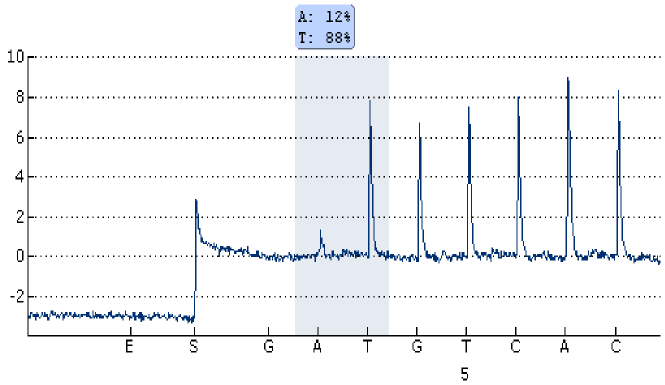

IGF2: Pancreas

Well: D4
Assay: IGF2
Sample ID: M1_P
Sequence to analyze: WGTCACAATTGGAGAACCCACATTGG

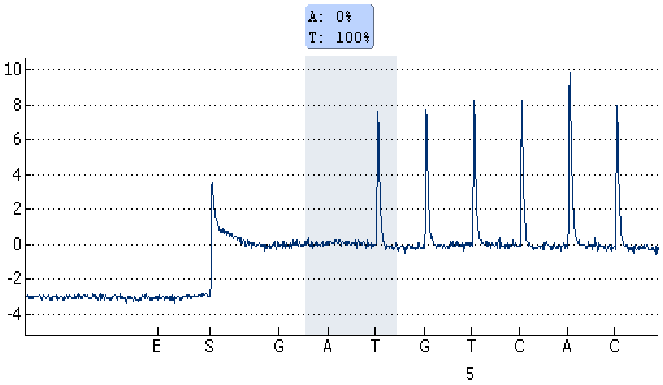

Well: D5
Assay: IGF2
Sample ID: M2_P
Sequence to analyze: WGTCACAATTGGAGAACCCACATTGG

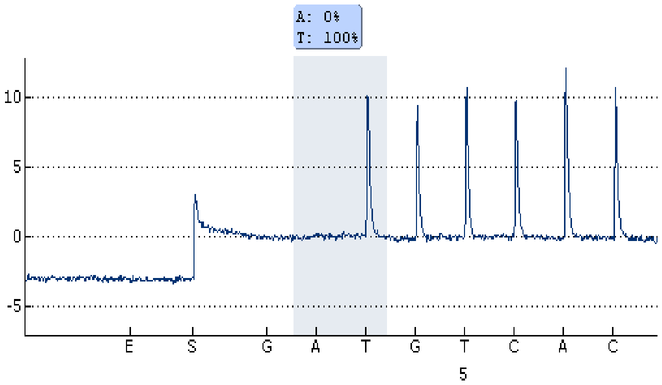

IGF2: Testes

Well: D7
Assay: IGF2
Sample ID: M1_T
Sequence to analyze: WGTCACAATTGGAGAACCCACATTGG

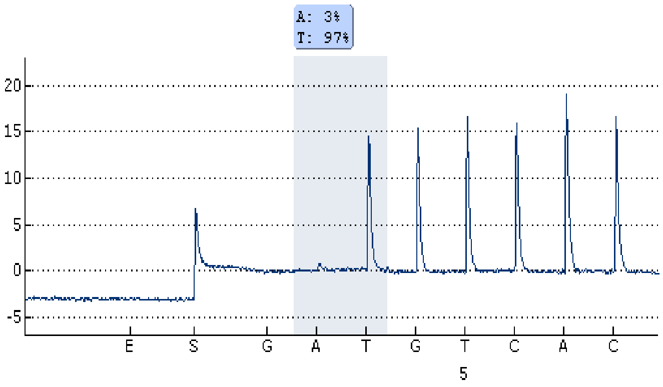

H19
Allele Specific Expression - cDNA
Qiagen Pyrosequencing

H19: Liver

Well: G7
Assay: H19_ASE only
Sample ID: F5_L
Sequence to analyze: CARGTAGTGCAGTGGTTGTAAAGTGCAG

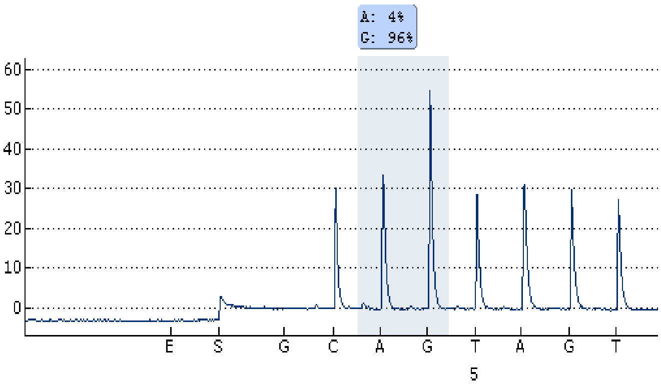

Well: G8
Assay: H19_ASE only
Sample ID: F6_L
Sequence to analyze: CARGTAGTGCAGTGGTTGTAAAGTGCAG

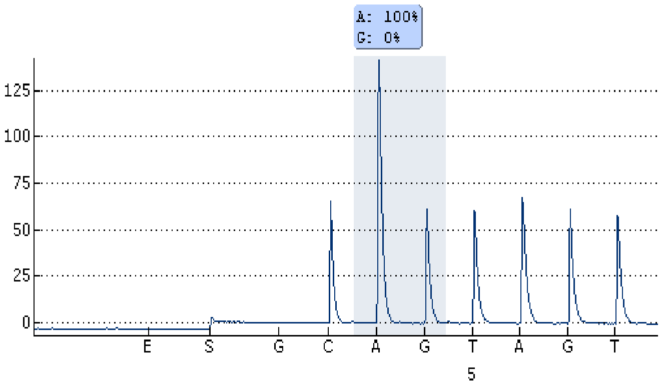

Well: G9
Assay: H19_ASE only
Sample ID: M1_L
Sequence to analyze: CARGTAGTGCAGTGGTTGTAAAGTGCAG

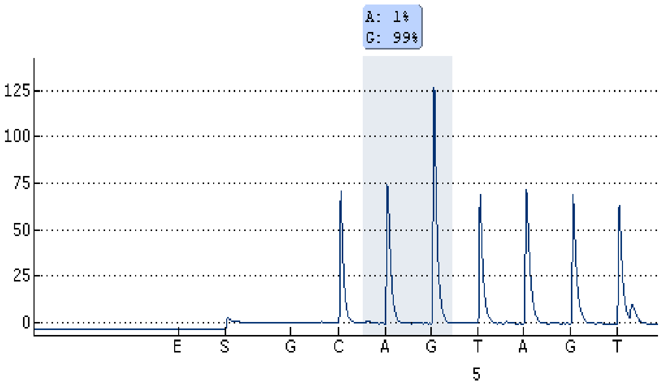

Well: G10
Assay: H19_ASE only
Sample ID: M4_L
Sequence to analyze: CARGTAGTGCAGTGGTTGTAAAGTGCAG

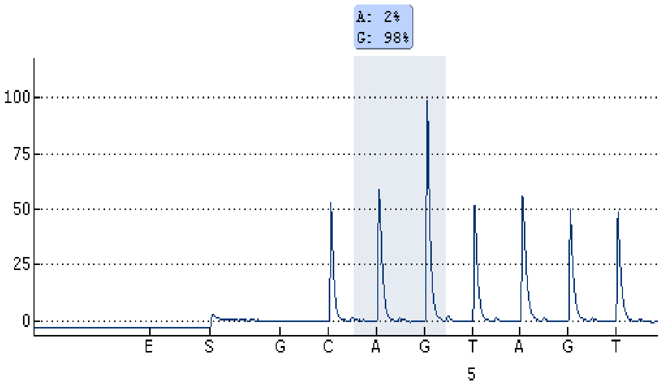

H19: Kidney

Well: H2
Assay: H19_ASE only
Sample ID: F6_K
Sequence to analyze: CARGTAGTGCAGTGGTTGTAAAGTGCAG

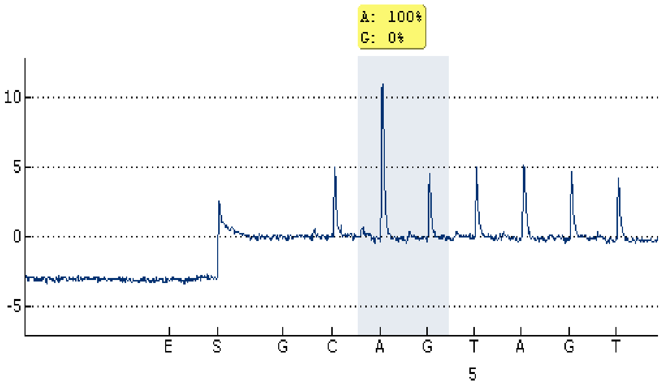

Well: H3
Assay: H19_ASE only
Sample ID: M1_K
Sequence to analyze: CARGTAGTGCAGTGGTTGTAAAGTGCAG

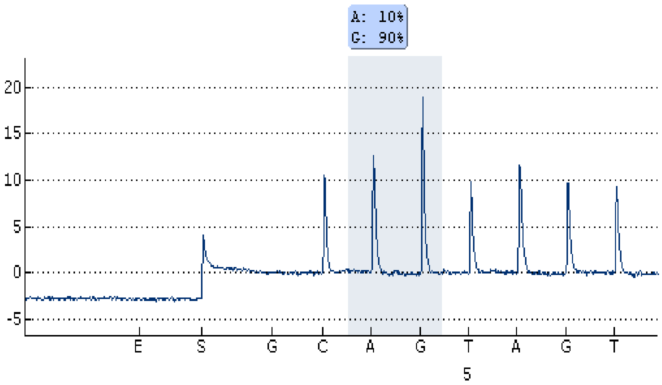

Well: H4
Assay: H19_ASE only
Sample ID: M2_K
Sequence to analyze: CARGTAGTGCAGTGGTTGTAAAGTGCAG

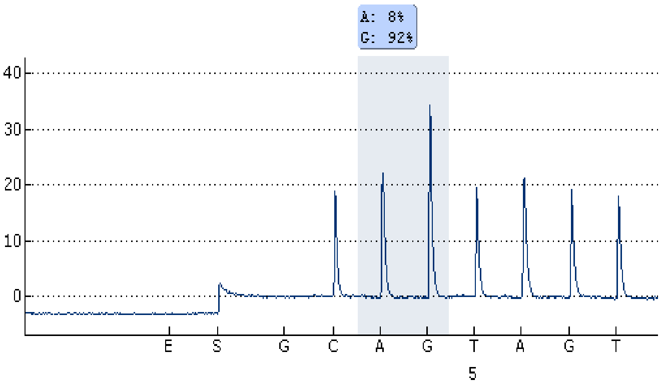

H19: Pancreas

Well: H5
Assay: H19_ASE only
Sample ID: F5_P
Sequence to analyze: CARGTAGTGCAGTGGTTGTAAAGTGCAG

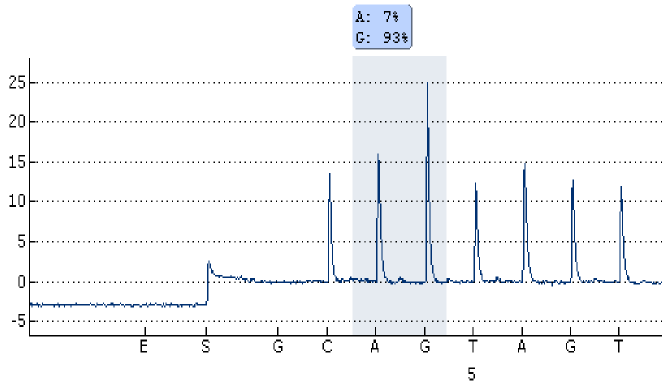

Well: H6
Assay: H19_ASE only
Sample ID: F6_P
Sequence to analyze: CARGTAGTGCAGTGGTTGTAAAGTGCAG

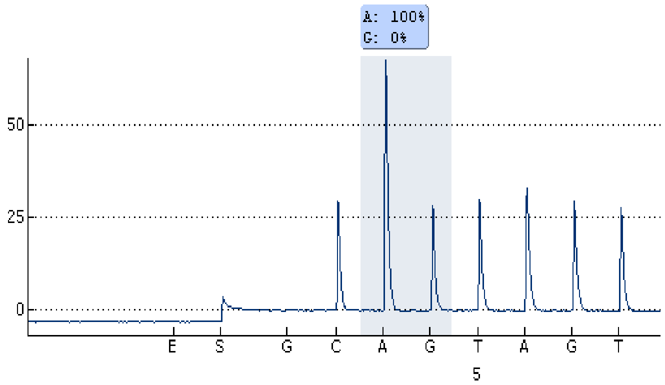

H19: Testes

Well: H7
Assay: H19_ASE only
Sample ID: M1_T
Sequence to analyze: CARGTAGTGCAGTGGTTGTAAAGTGCAG

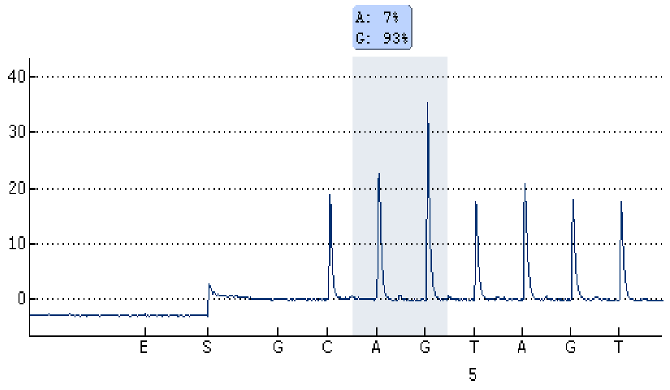

DLK1
Allele Specific Expression - cDNA
Qiagen Pyrosequencing

DLK1: Liver

Well: E5
Assay: DLK1_ASE
Sample ID: F4_L
Sequence to analyze: AYGATGGCCTCTATGAATGCTCCTGTG

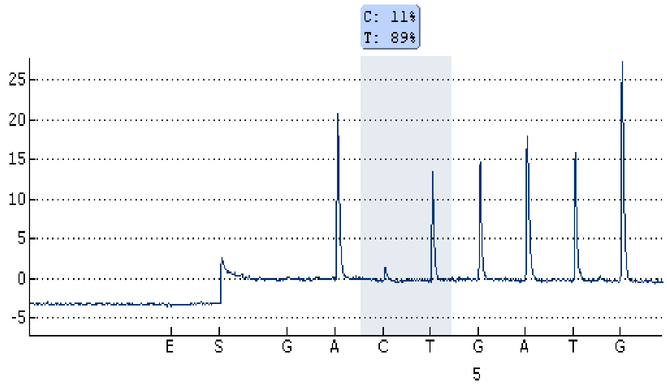

Well: E6
Assay: DLK1_ASE
Sample ID: F5_L
Sequence to analyze: AYGATGGCCTCTATGAATGCTCCTGTG

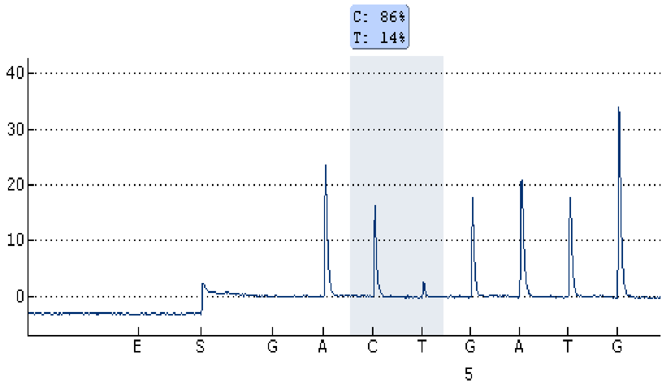

Well: E7
Assay: DLK1_ASE
Sample ID: M3_L
Sequence to analyze: AYGATGGCCTCTATGAATGCTCCTGTG

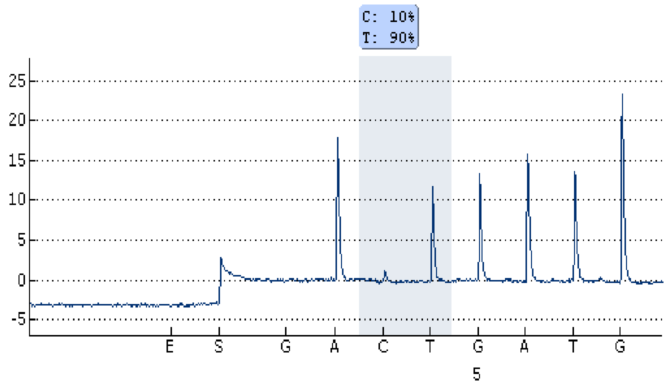

Well: E8
Assay: DLK1_ASE
Sample ID: M4_L
Sequence to analyze: AYGATGGCCTCTATGAATGCTCCTGTG

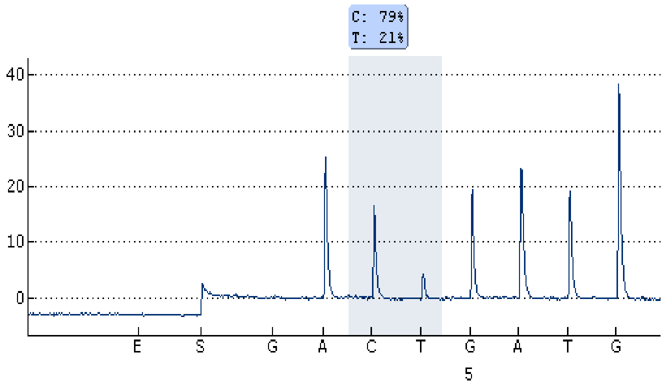

DLK1: Kidney

Well: E9
Assay: DLK1_ASE
Sample ID: F4_K
Sequence to analyze: AYGATGGCCTCTATGAATGCTCCTGTG

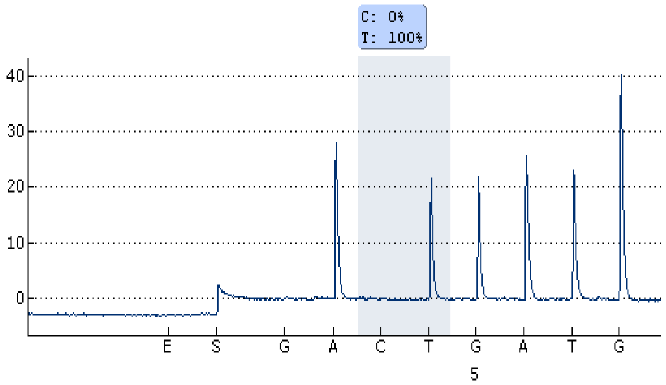

Well: E10
Assay: DLK1_ASE
Sample ID: F5_K
Sequence to analyze: AYGATGGCCTCTATGAATGCTCCTGTG

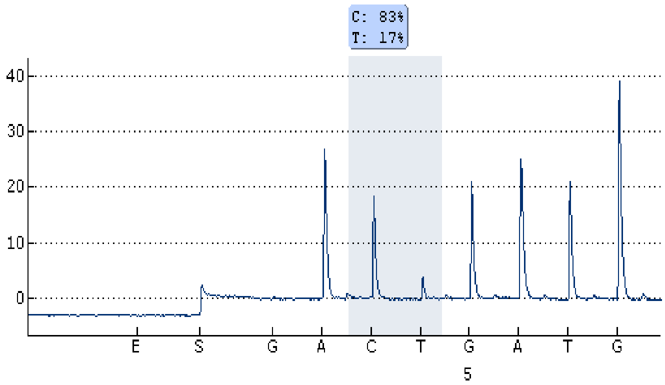

Well: E11
Assay: DLK1_ASE
Sample ID: M3_K
Sequence to analyze: AYGATGGCCTCTATGAATGCTCCTGTG

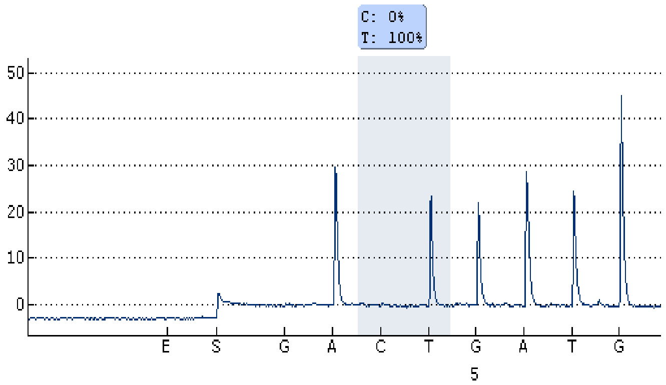

Well: E12
Assay: DLK1_ASE
Sample ID: M4_K
Sequence to analyze: AYGATGGCCTCTATGAATGCTCCTGTG

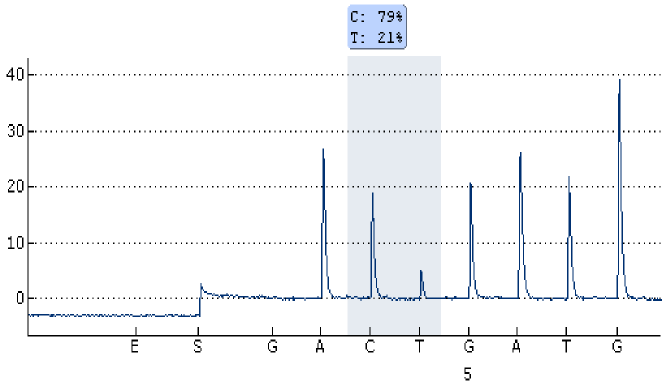

DLK1: Pancreas

Well: F1
Assay: DLK1_ASE
Sample ID: F4_P
Sequence to analyze: AYGATGGCCTCTATGAATGCTCCTGTG

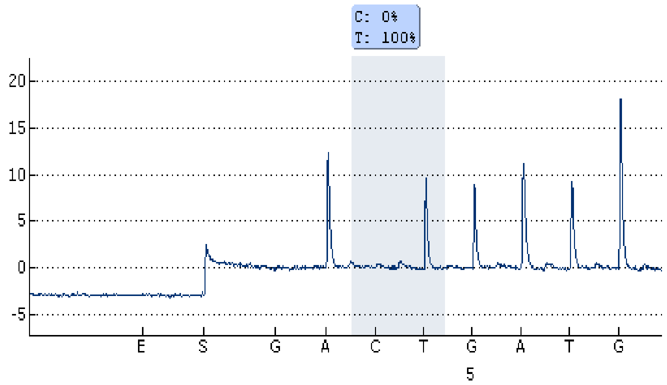

Well: F2
Assay: DLK1_ASE
Sample ID: F5_P
Sequence to analyze: AYGATGGCCTCTATGAATGCTCCTGTG

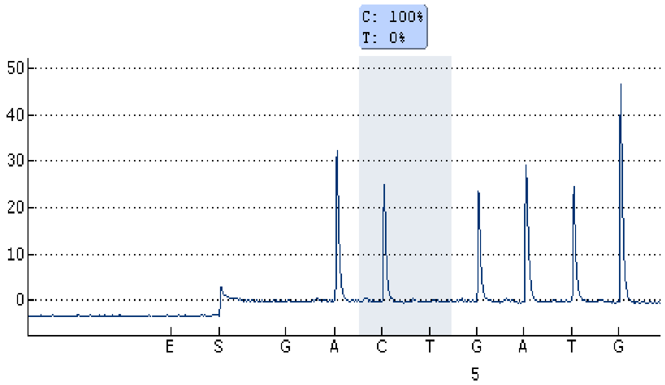

Well: F3
Assay: DLK1_ASE
Sample ID: M3_P
Sequence to analyze: AYGATGGCCTCTATGAATGCTCCTGTG

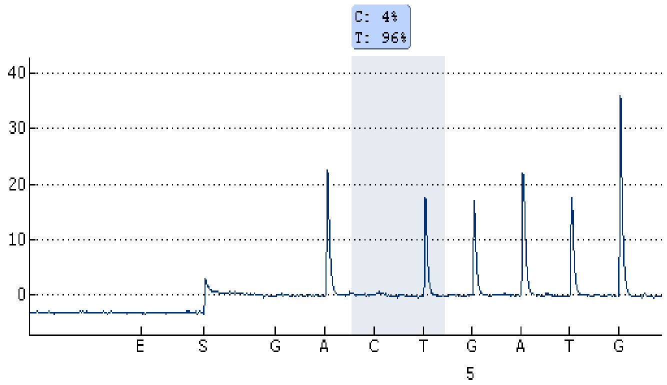

DLK1: Testes

Well: F5
Assay: DLK1_ASE
Sample ID: M3_T
Sequence to analyze: AYGATGGCCTCTATGAATGCTCCTGTG

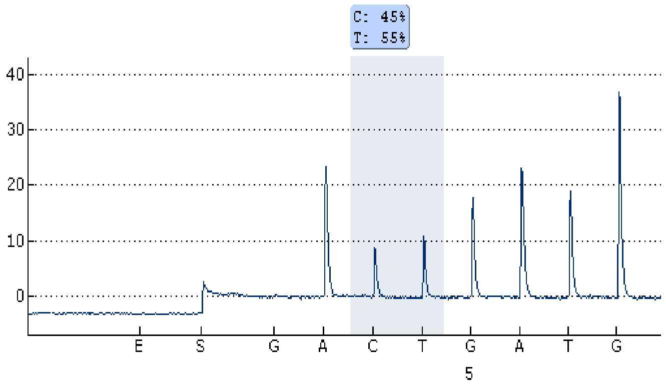

PEG3
Allele Specific Expression - cDNA
Qiagen Pyrosequencing

PEG3: Liver

Well: C5
Assay: PEG3_ASE
Sample ID: F6_L
Sequence to analyze: GGAYGTTTCATTGCACAAGAGGGAGTCAG

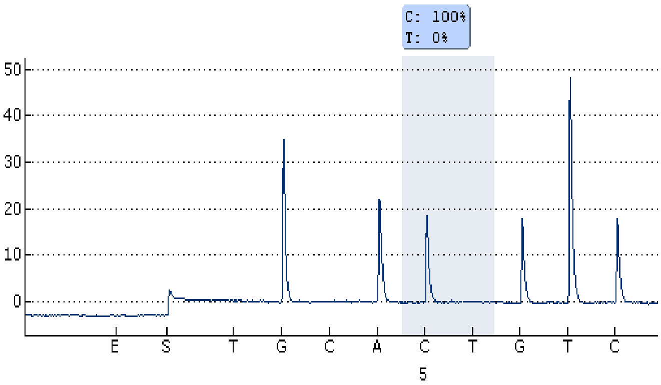

Well: C6
Assay: PEG3_ASE
Sample ID: M1_L
Sequence to analyze: GGAYGTTTCATTGCACAAGAGGGAGTCAG

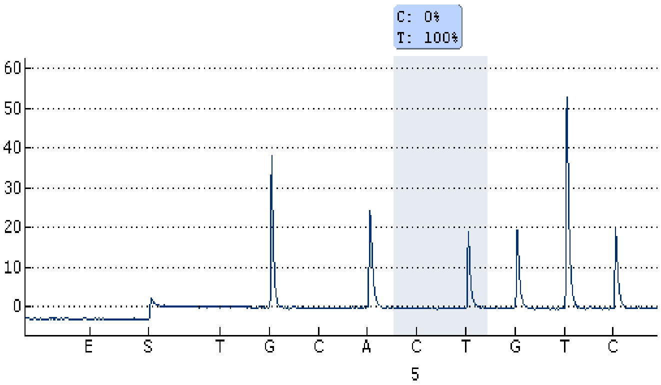

Well: C7
Assay: PEG3_ASE
Sample ID: M3_L
Sequence to analyze: GGAYGTTTCATTGCACAAGAGGGAGTCAG

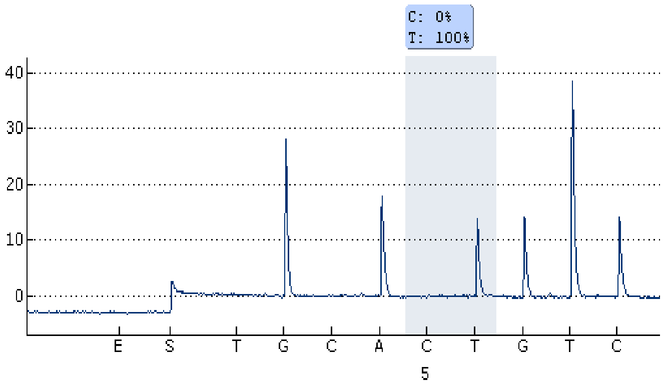

PEG3: Kidney

Well: C8
Assay: PEG3_ASE
Sample ID: F6_K
Sequence to analyze: GGAYGTTTCATTGCACAAGAGGGAGTCAG

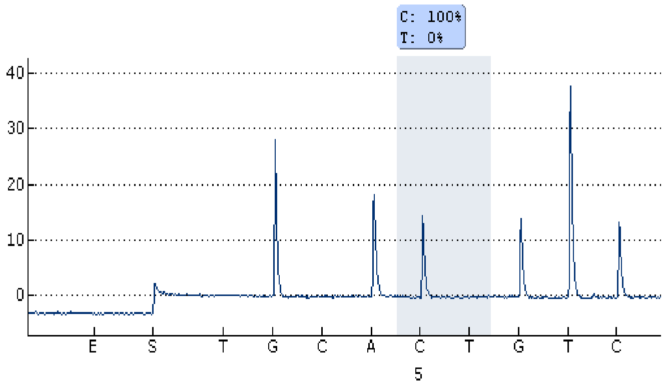

Well: C9
Assay: PEG3_ASE
Sample ID: M1_K
Sequence to analyze: GGAYGTTTCATTGCACAAGAGGGAGTCAG

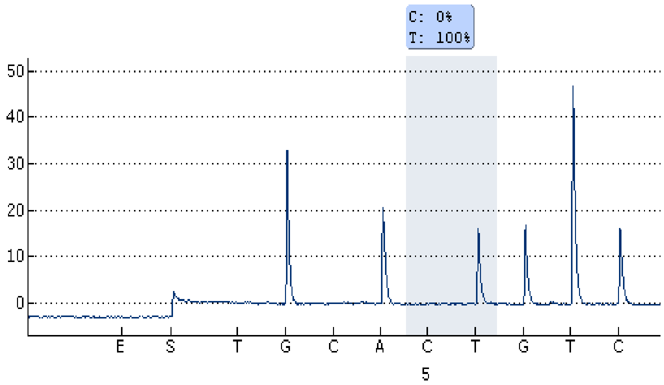

Well: C10
Assay: PEG3_ASE
Sample ID: M3_K
Sequence to analyze: GGAYGTTTCATTGCACAAGAGGGAGTCAG

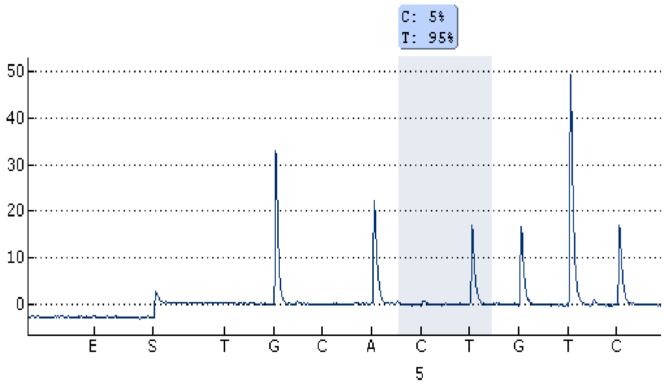

PEG3: Pancreas

Well: C11
Assay: PEG3_ASE
Sample ID: F6_P
Sequence to analyze: GGAYGTTTCATTGCACAAGAGGGAGTCAG

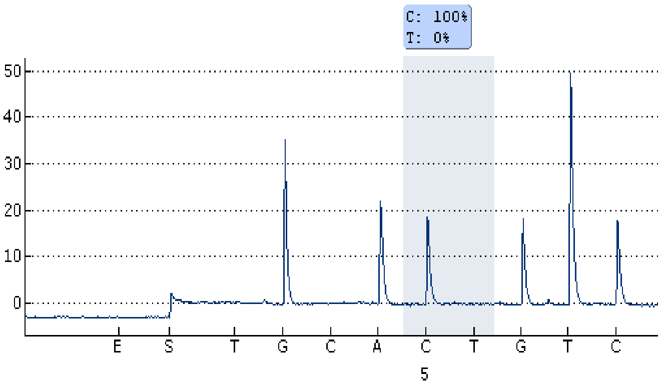

Well: C12
Assay: PEG3_ASE
Sample ID: M1_P
Sequence to analyze: GGAYGTTTCATTGCACAAGAGGGAGTCAG

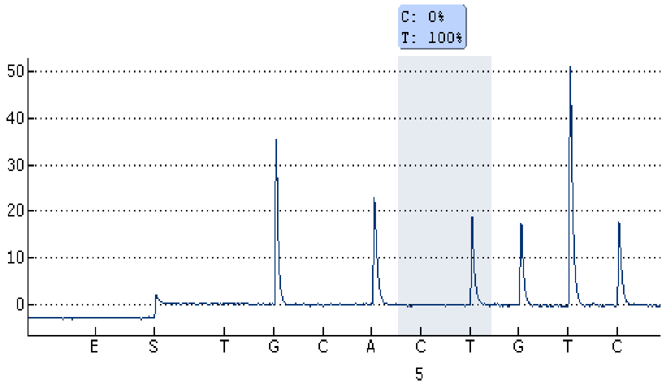

Well: D1
Assay: PEG3_ASE
Sample ID: M3_P
Sequence to analyze: GGAYGTTTCATTGCACAAGAGGGAGTCAG

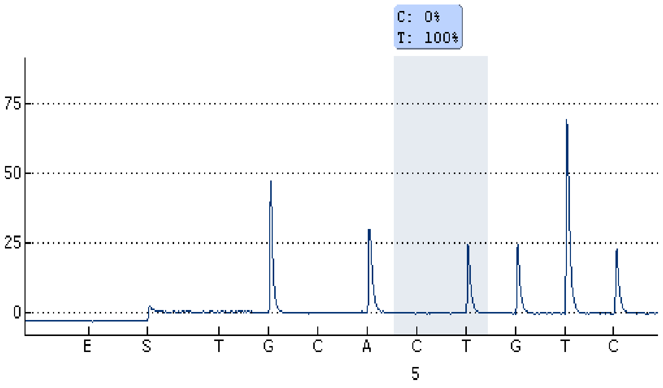

PEG3: Testes

Well: D2
Assay: PEG3_ASE
Sample ID: M1_T
Sequence to analyze: GGAYGTTTCATTGCACAAGAGGGAGTCAG

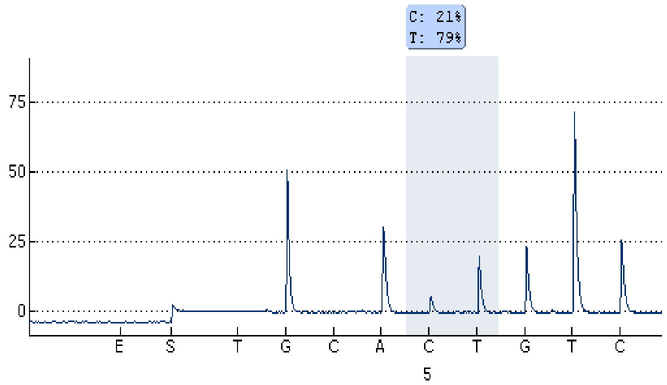

Well: D3
Assay: PEG3_ASE
Sample ID: M3_T
Sequence to analyze: GGAYGTTTCATTGCACAAGAGGGAGTCAG

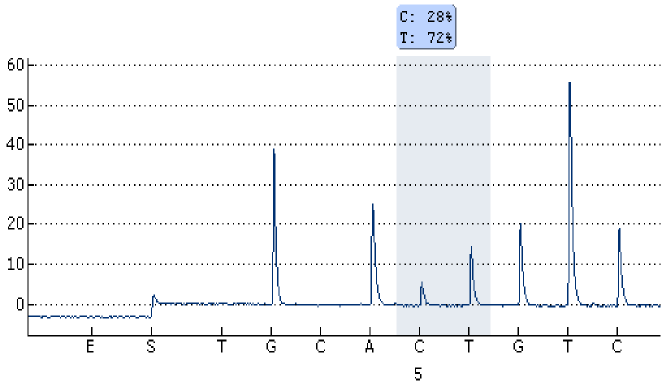

IGF2R
Allele Specific Expression - cDNA
Qiagen Pyrosequencing

IGF2R: Liver

Well: G6
Assay: IGF2R_Rhesus1
Sample ID: F5_L
Sequence to analyze:
[A/C/G]GAGGTGAAAGTTCACTCGGGCAGGGGA[G/A]CTGGGGCAGAG
AGCTCCCACCCACT

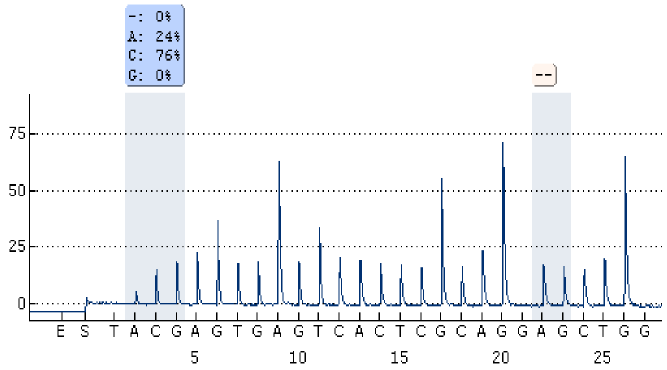

Well: G7
Assay: IGF2R_Rhesus1
Sample ID: F6_L
Sequence to analyze:
[A/C/G]GAGGTGAAAGTTCACTCGGGCAGGGGA[G/A]CTGGGGCAGAG
AGCTCCCACCCACTA

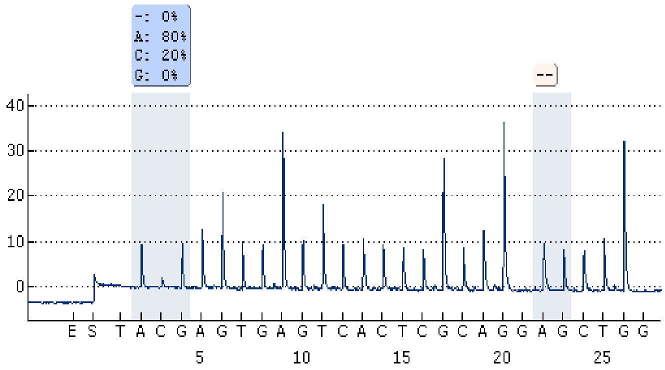

Well: G8
Assay: IGF2R_Rhesus1
Sample ID: M1_L
Sequence to analyze:
[A/C/G]GAGGTGAAAGTTCACTCGGGCAGGGGA[G/A]CTGGGGCAGAG
AGCTCCCACCCACT

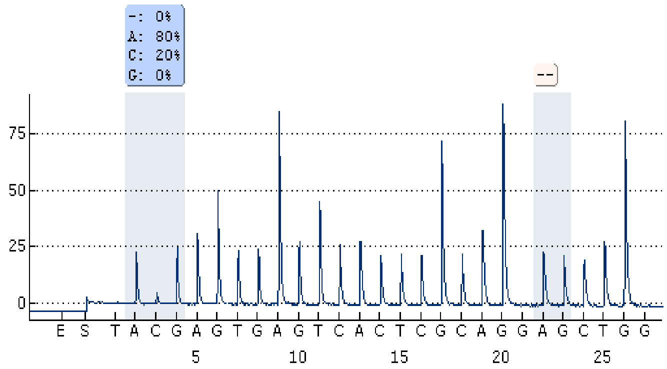

Well: G9
Assay: IGF2R_Rhesus1
Sample ID: M2_L
Sequence to analyze:
[A/C/G]GAGGTGAAAGTTCACTCGGGCAGGGGA[G/A]CTGGGGCAGAG
AGCTCCCACCCACT

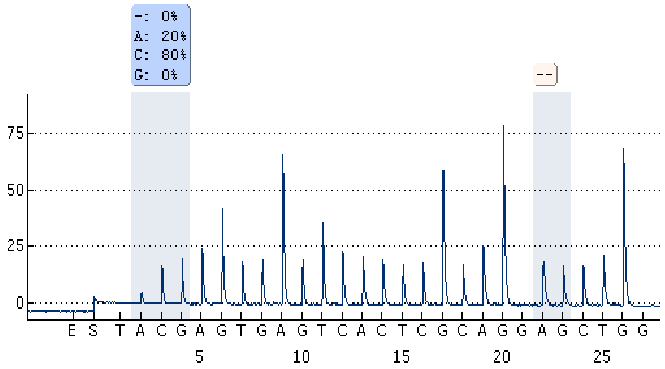

IGF2R: Kidney

Well: G10
Assay: IGF2R_Rhesus1
Sample ID: F5_K
Sequence to analyze:
[A/C/G]GAGGTGAAAGTTCACTCGGGCAGGGGA[G/A]CTGGGGCAGAG
AGCTCCCACCCACT

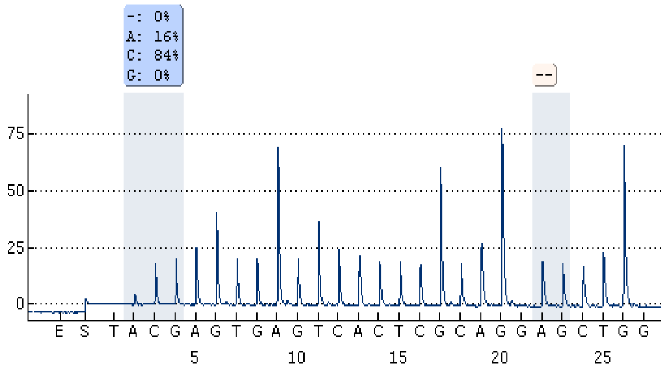

Well: G11
Assay: IGF2R_Rhesus1
Sample ID: F6_K
Sequence to analyze:
[A/C/G]GAGGTGAAAGTTCACTCGGGCAGGGGA[G/A]CTGGGGCAGAG
AGCTCCCACCCACT

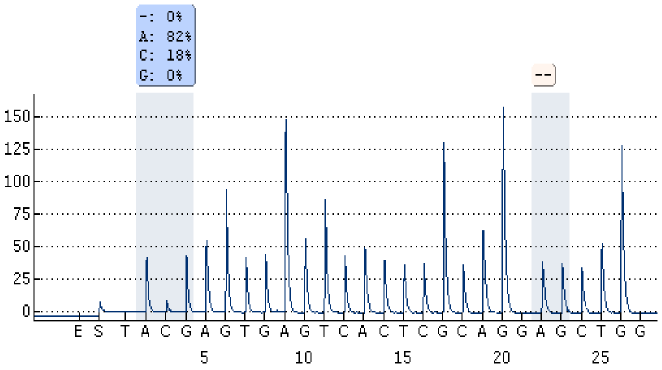

Well: G12
Assay: IGF2R_Rhesus1
Sample ID: M1_K
Sequence to analyze:
[A/C/G]GAGGTGAAAGTTCACTCGGGCAGGGGA[G/A]CTGGGGCAGAG
AGCTCCCACCCACT

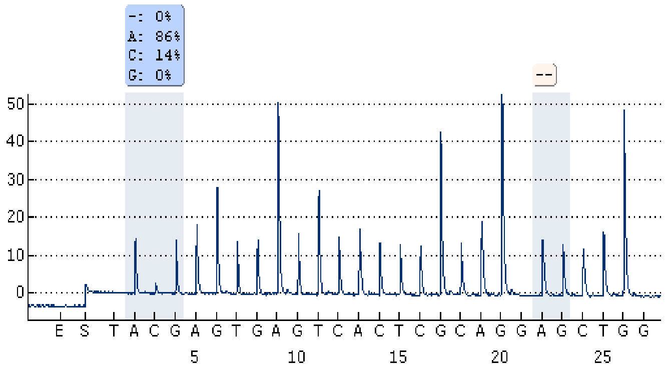

Well: H1
Assay: IGF2R_Rhesus1
Sample ID: M2_K
Sequence to analyze:
[A/C/G]GAGGTGAAAGTTCACTCGGGCAGGGGA[G/A]CTGGGGCAGAG
AGCTCCCACCCACT

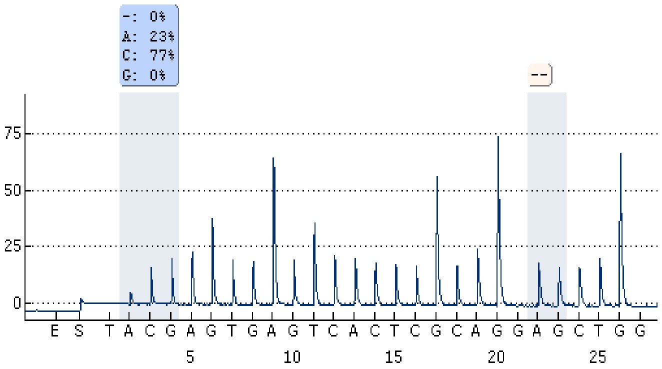

IGF2R: Pancreas

Well: H2
Assay: IGF2R_Rhesus1
Sample ID: F5_P
Sequence to analyze:
[A/C/G]GAGGTGAAAGTTCACTCGGGCAGGGGA[G/A]CTGGGGCAGAG
AGCTCCCACCCACT

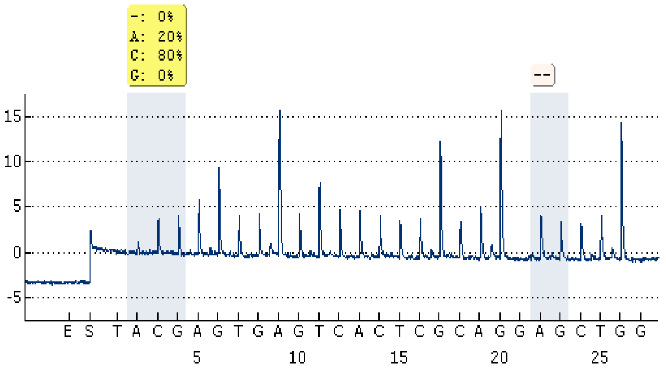

Well: H3
Assay: IGF2R_Rhesus1
Sample ID: F6_P
Sequence to analyze:
[A/C/G]GAGGTGAAAGTTCACTCGGGCAGGGGA[G/A]CTGGGGCAGAG
AGCTCCCACCCACT

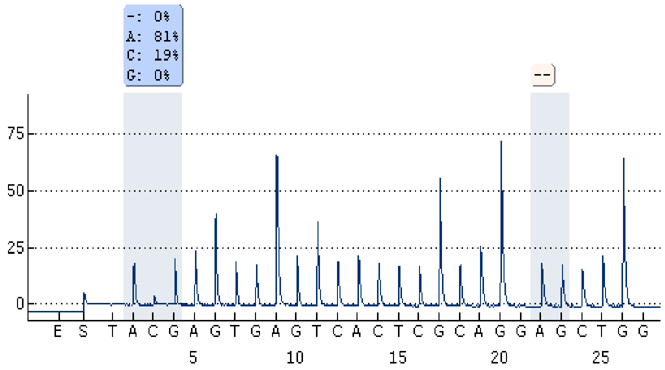

Well: H4
Assay: IGF2R_Rhesus1
Sample ID: M1_P
Sequence to analyze:
[A/C/G]GAGGTGAAAGTTCACTCGGGCAGGGGA[G/A]CTGGGGCAGAG
AGCTCCCACCCACT

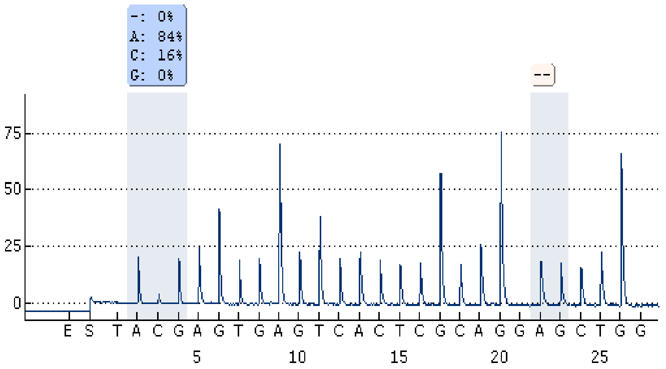

Well: H5
Assay: IGF2R_Rhesus1
Sample ID: M2_P
Sequence to analyze:
[A/C/G]GAGGTGAAAGTTCACTCGGGCAGGGGA[G/A]CTGGGGCAGAG
AGCTCCCACCCACT

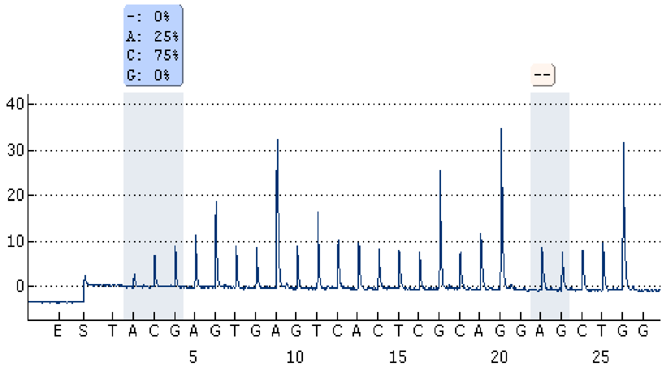

IGF2R: Testes

Well: H6
Assay: IGF2R_Rhesus1
Sample ID: M1_T
Sequence to analyze:
[A/C/G]GAGGTGAAAGTTCACTCGGGCAGGGGA[G/A]CTGGGGCAGAG
AGCTCCCACCCACT

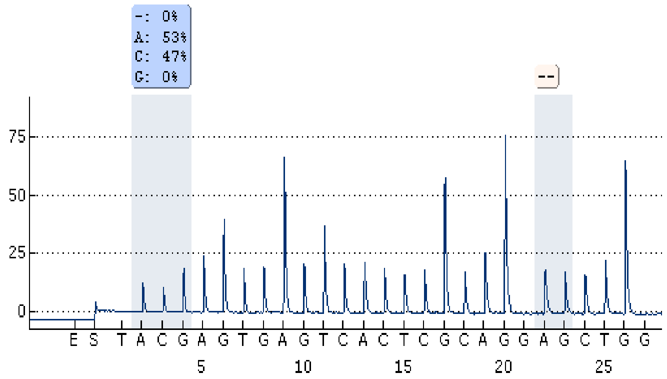

KCNQ1
Allele Specific Expression - cDNA
Qiagen Pyrosequencing

KCNQ1: Liver

Well: G11
Assay: KCNQ1_SNP2
Sample ID: F5_L
Sequence to analyze: [G/C]AGGGGCCCCGACGAGGGGTCCTGAT

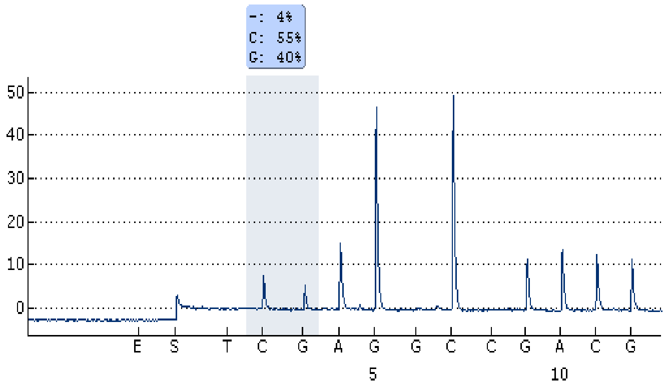

Well: H2
Assay: KCNQ1_SNP2
Sample ID: M2_L
Sequence to analyze: [G/C]AGGGGCCCCGACGAGGGGTCCTGAT

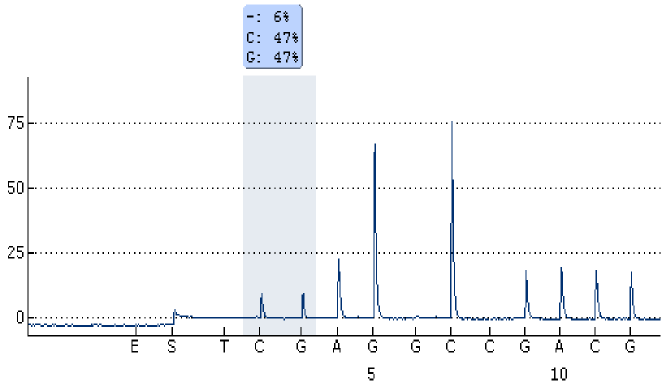

KCNQ1: Kidney

Well: H3
Assay: KCNQ1_SNP2
Sample ID: F5_K
Sequence to analyze: [G/C]AGGGGCCCCGACGAGGGGTCCTGAT

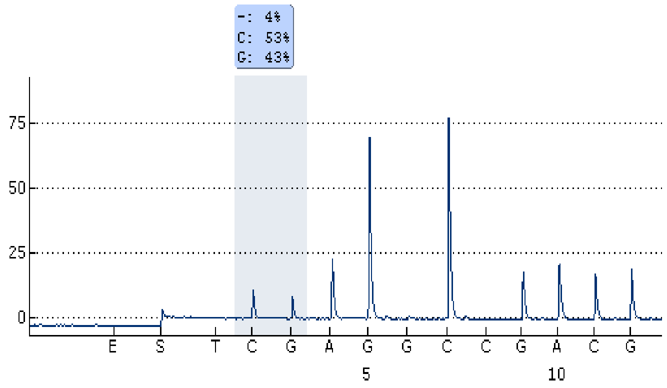

Well: H4
Assay: KCNQ1_SNP2
Sample ID: F6_K
Sequence to analyze: [G/C]AGGGGCCCCGACGAGGGGTCCTGAT

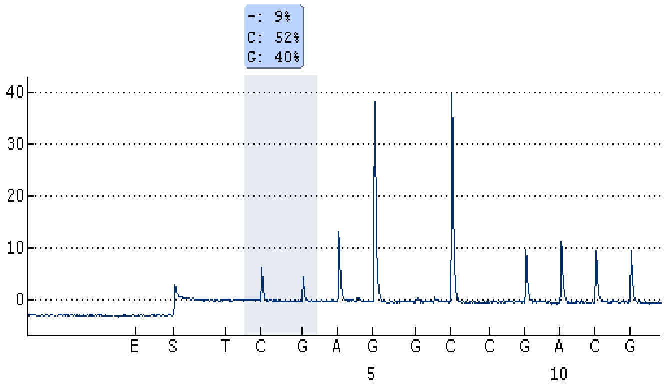

Well: H6
Assay: KCNQ1_SNP2
Sample ID: M2_K
Sequence to analyze: [G/C]AGGGGCCCCGACGAGGGGTCCTGAT

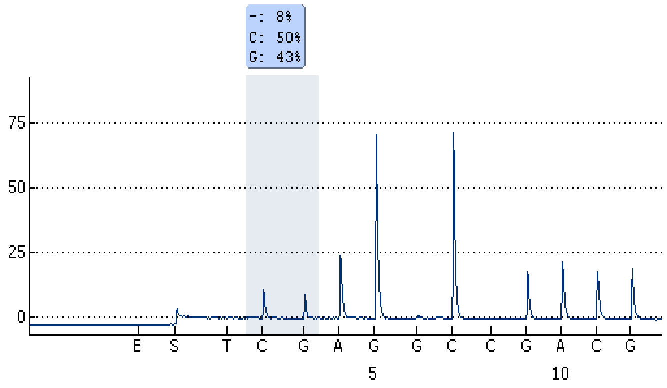

KCNQ1: Pancreas

Well: H7
Assay: KCNQ1_SNP2
Sample ID: F5_P
Sequence to analyze: [G/C]AGGGGCCCCGACGAGGGGTCCTGAT

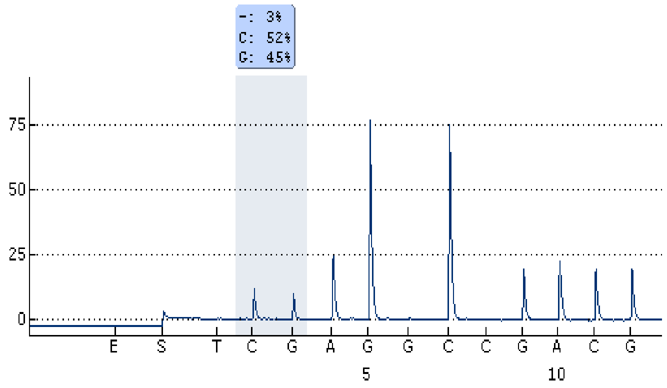

Well: H9
Assay: KCNQ1_SNP2
Sample ID: M1_P
Sequence to analyze: [G/C]AGGGGCCCCGACGAGGGGTCCTGAT

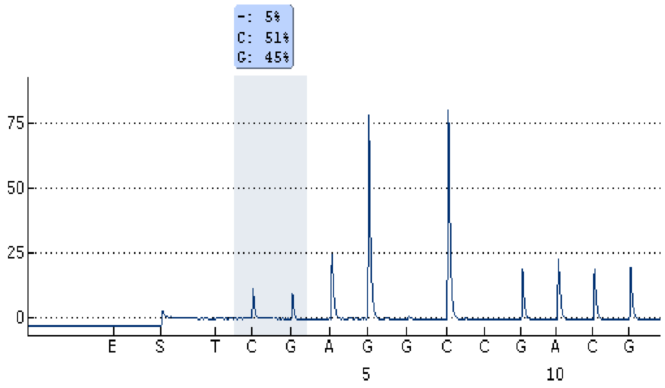

Well: H10
Assay: KCNQ1_SNP2
Sample ID: M2_P
Sequence to analyze: [G/C]AGGGGCCCCGACGAGGGGTCCTGAT

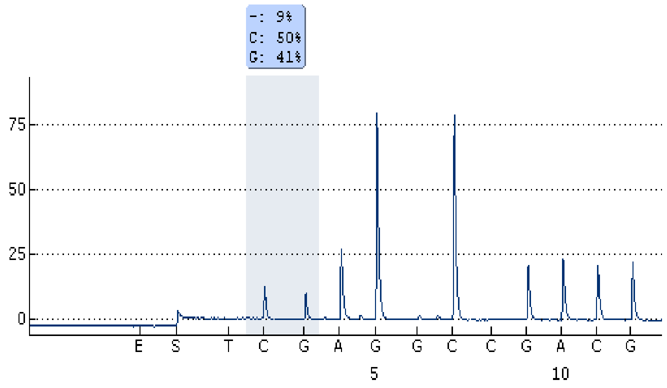

KCNQ1: Testes

Well: H11
Assay: KCNQ1_SNP2
Sample ID: M1_T
Sequence to analyze: [G/C]AGGGGCCCCGACGAGGGGTCCTGAT

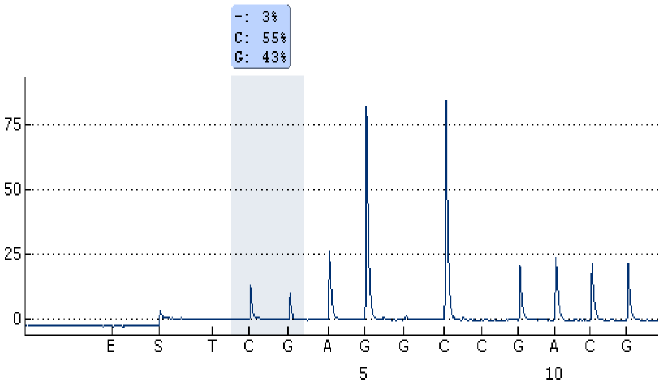

KCNQ1: Placenta

Well: B2
Assay: KCNQ1_1
Sample ID: #24
Sequence to analyze: [G/C]AGGGGCCCCGACGAGGGGTCCTGAT

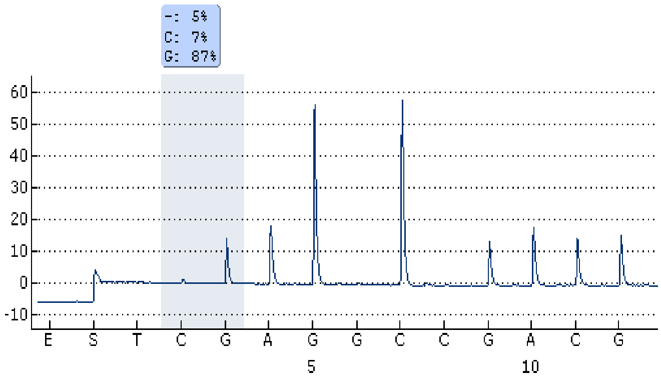

Well: B3
Assay: KCNQ1_1
Sample ID: #33
Sequence to analyze: [G/C]AGGGGCCCCGACGAGGGGTCCTGAT

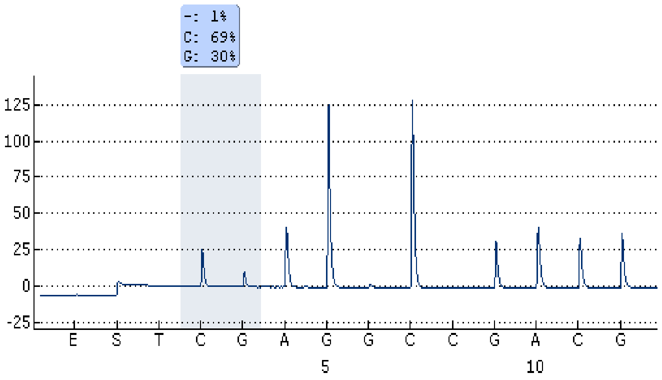

KCNQ1: Umbilical Cord

Well: B5
Assay: KCNQ1_1
Sample ID: #24
Sequence to analyze: [G/C]AGGGGCCCCGACGAGGGGTCCTGAT

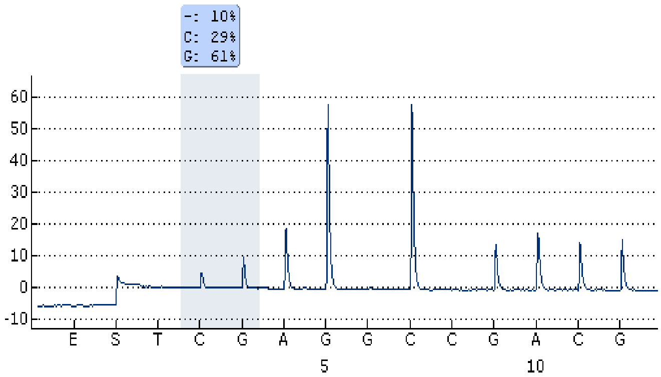

Well: B6
Assay: KCNQ1_1
Sample ID: #33
Sequence to analyze: [G/C]AGGGGCCCCGACGAGGGGTCCTGAT

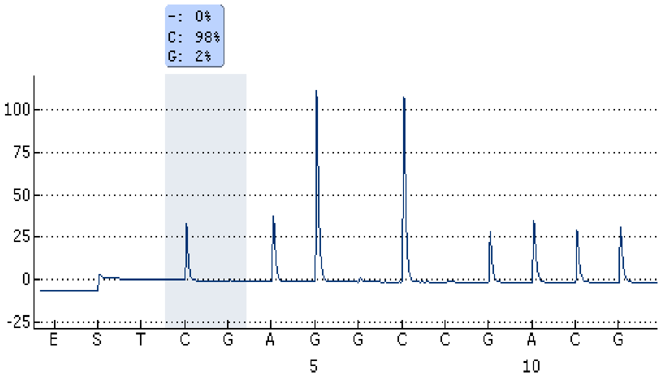

Supplement: Supplemental Material [file supp_gr.183301.114_Supplemental_Data.pdf]
