# Supplementary material for: Germline and somatic imprinting in the nonhuman primate highlights species differences in oocyte methylation
Source: Genome Res. 2015 May;25(5):611–23. doi: 10.1101/gr.183301.114 (PMC4417110; doi:10.1101/gr.183301.114)
Supplement: Supplemental Material [file supp_gr.183301.114_Supplemental_Information.docx]

**SUPPLEMENTARY FILE 1**

**Germline and Somatic Imprinting in the Non-Human Primate Uncovers Primate-Specific Acquisition**

Clara Y. Cheong^1^, Keefe Chng^1,^ ^•^, Shilen Ng^1,•^, Siew Boom Chew^1, •^, Louiza Chan^1^, Anne C Ferguson-Smith^1,2^

^1^Growth, Development and Metabolism Program, Singapore Institute for Clinical Sciences, Agency for Science, Technology and Research (A-STAR), 30 Medical Drive, Singapore 117609

^2^ Department of Genetics, University of Cambridge, Downing Street, Cambridge CB2 3EH, UK

• Present affiliations; KC: Crown Bioscience Inc., 3375 Scott Blvd., Suite 108, Santa Clara, CA 95054 ; SN: Health Sciences Authority, 3 Biopolis Drive, Synapse, Singapore 138623; SBC: Syngenta APAC Pte Ltd, 50 Science Park Road, #06-01, The Kendall, Singapore 117406

**Supplementary Table 1:** Allelic Expression by NHP Tissue and Gene

**Supplementary Figure 2:** Pyrosequencing and Capillary Sequencing Data

Supplementary Figure 3: Imprinted Gene Cluster Maps and Putative Cynomolgus DMRs

**Supplementary Figure 4:** Novel Variant of CDKN1C in Cynomolgus Macaque

**Supplementary Figure 5:** Additional Bisulfite Diagrams of Macaque Samples

**Supplementary Table 6:** Known Parental Origin of Germline Methylation

Supplementary Table 7: Primers for Bisulfite DNA Amplification & Sequencing

Supplementary Table 8: Primers for Pyrosequencing of Bisulfite-converted DNA

**Supplementary Table 9:** Primers for Genotyping and RT-PCR

**Supplementary Table 10:** List of Identified Cynomolgus SNPs

**Cheong_Supplementary Table 1**

| **Locus** | **Gene** | **Known Allelic Exp in Human** | **Allelic Expression** | | | | | | | | | | | | | | | | | |
| --- | --- | --- | --- | --- | --- | --- | --- | --- | --- | --- | --- | --- | --- | --- | --- | --- | --- | --- | --- | --- |
|  |  |  | **Placenta** | | **Umbilical Cord** | | **Muscle** | | **Liver** | | **Kidney** | | **Pancreas** | | **Testes** | | **Cerebellum** | | **Blood** | |
|  |  |  | **B** | **M** | **B** | **M** | **B** | **M** | **B** | **M** | **B** | **M** | **B** | **M** | **B** | **M** | **B** | **M** | **B** | **M** |
| ***PLAGL1*** | *PLAGL1* | *P* | 0 | 5 | 0 | 7♦ | 0 | 5 | 0 | 4♦ | 0 | 3 | 0 | 3 | no SNP | | 0 | 2 | - | - |
| ***IGF2R*** | *IGF2R* | *M/=* | 0 | 3♦ | 0 | 3♦ | 0 | 5♦ | 0 | 6♦ | 0 | 5♦ | 0 | 4♦ | 1 | 0 | - | - | 4 | 0 |
|  | Putative *AIRN* | *P* | no SNP | | no SNP | | 0 | 7 | 1 | 5 | 0 | 6 | - | - | - | - | - | - | - | - |
|  | *SLC22A1* | *=* | 0 | 3 | 0 | 3 | 3 | 0 | 5 | 0 | 4 | 1 | - | - | - | - | - | - | - | - |
|  | *SLC22A2* | *M* | 2 | 0 | 2 | 0 | 4 | 0 | 4 | 0 | 3 | 0 | 2 | 0 | 1 | 0 | - | - | - | - |
|  | *SLC22A3* | *M* | 3 | 1 | 5 | 0 | 1 | 1 | 1 | 1 | 1 | 0 | 1 | 0 | 1 | 0 | - | - | - | - |
| ***INPP5F*** | *INPP5F* transcript var. 1 | *=* | 6 | 0 | 4 | 0 | 1 | 0 | - | - | - | - | - | - | - | - | - | - | - | - |
|  | *INPP5F* transcript var. 2 | *P* | 1 | 2♦ | 0 | 4 | 0 | 2 | 0 | 1 | 0 | 1 | - | - | - | - | - | - | - | - |
| ***IGF2*** | *IGF2* | *P* | 0 | 2 | 0 | 2 | 1 | 1 | 3 | 0 | 1 | 2♦ | 0 | 2 | 0 | 1 | - | - | - | - |
|  | *H19* | *M* | 0 | 3 | 0 | 3 | 0 | 2 | 0 | 3 | 0 | 2 | 0 | 2 | 0 | 1 | 0 | 1 | - | - |
|  | *INS* | *P* | 0 | 2 | 0 | 2 | no SNP | | no SNP | | no SNP | | no SNP | | no SNP | | no SNP | | no SNP | |
| ***KCNQ1*** | *KCNQ1* | *M* | 1 | 4♦ | 2 | 2♦ | 3 | 0 | 6 | 0 | 5 | 0 | 3 | 0 | 1 | 0 | - | - | 2 | 0 |
|  | *CDKN1C* | *M* | no SNP | | no SNP | | 0 | 4 | 0 | 4 | 0 | 4 | - | - | - | - | no SNP | | - | - |
|  | *SLC22A18* | *M* | 0 | 5 | 0 | 4 | 5 | 0 | 5 | 0 | 4 | 0 | - | - | - | - | 1 | 0 | - | - |
| ***DLK1*** | *DLK1* | *P* | 0 | 2 | no SNP | | 0 | 4 | 0 | 4♦ | 0 | 4♦ | 0 | 4 | 0 | 1 | no SNP | | 0 | 3 |
|  | *MEG3* | *M* | 0 | 5 | 0 | 5 | no SNP | | no SNP | | no SNP | | no SNP | | no SNP | | no SNP | | no SNP | |
|  | *DIO3* | *P* | 2 | 1 | 3 | 0 | 0 | 4♦ | 0 | 3♦ | 3 | 1 | 0 | 3♦ | - | - | no SNP | | 4 | 0 |
| ***SNRPN*** | *MKRN3* | *P* | 4 | 0 | 0 | 4 | 0 | 5 | - | - | - | - | - | - | 1 | 0 | 0 | 3 | - | - |
|  | *MAGEL2* | *P* | 4 | 2 | 0 | 6 | 0 | 3 | - | - | - | - | - | - | - | - | 0 | 2 | - | - |
|  | *NDN* | *P* | 4 | 1 | 0 | 6 | 0 | 4 | - | - | - | - | - | - | 0 | 2 | 0 | 2 | - | - |
|  | *SNRPN* | *P* | 0 | 6 | 0 | 6 | 0 | 6 | - | - | - | - | - | - | 0 | 3 | 0 | 4 | - | - |
|  | *UBE3A* | *M* | 3 | 0 | 3 | 0 | 6 | 0 | - | - | - | - | - | - | 2 | 0 | 1 | 1 | - | - |
|  | *ATP10A* | *M* | 2 | 3 | 5 | 0 | 7 | 0 | - | - | - | - | - | - | 2 | 0 | 4 | 0 | - | - |
| ***PEG3*** | *PEG3* | *P* | 0 | 3 | 0 | 3 | - | - | 0 | 2 | 0 | 3 | 0 | 3 | 0 | 2♦ | no SNP | | - | - |
|  | *ZIM2* | *P* | 3 | 2 | 0 | 4 | - | - | - | - | 0 | 4 | 0 | 4 | 2 | 0 | no SNP | | - | - |
|  | *USP29* | *P* | 0 | 2 | - | - | - | - | - | - | 0 | 2 | - | - | 2 | 0 | 0 | 1 | - | - |
|  | *ZIM3* | *M* | 0 | 2 | 0 | 1 | - | - | - | - | - | - | 0 | 1 | 1 | 0 | - | - | - | - |
|  | *ZNF264* | *P* | 0 | 5 | 0 | 3 | - | - | - | - | 5 | 0 | 5 | 0 | 2 | 0 | - | - | - | - |
| ***L3MBTL1*** | *L3MBTL1* | *P* | 0 | 3♦ | 1 | 1 | 4 | 0 | 0 | 6 | 0 | 6 | 3 | 3 | 2 | 0 | no SNP | | 1 | 3 |
|  | *SGK2* | *P* | 1 | 2 | 3 | 1 | 5 | 0 | 6 | 0 | 6 | 0 | 2 | 0 | 3 | 0 | no SNP | | 2 | 2 |
|  | *GDAP1L1* | *P* | no SNP | | no SNP | | 3 | 0 | 4 | 0 | 3 | 0 | 2 | 0 | 1 | 0 | 1 | 0 | - | - |
| ***NAP1L5*** | *NAP1L5* | *P* | 0 | 3♦ | 3 | 0 | 2 | 0 | 1 | 1♦ | 1 | 0 | 1 | 0 | 1 | 0 | - | - | - | - |
| ***COMMD1*** | *COMMD1* | *U* | 2 | 0 | 3 | 0 | 2 | 0 | 3 | 0 | 3 | 0 | - | - | 2 | 0 | no SNP | | - | - |

## Supplementary Table 1: Allelic Expression by NHP Tissue and Gene

In tissue-specific data columns, the number of informative macaque samples with mono (M) or biallelic (B) expression of imprinted genes are shown.

No SNP: no informative samples; “♦” indicates that some or all samples showed incomplete imprinted expression, with the minor allele expressed at levels up to 30% of the primary expressed allele, as determined by sequencing chromatogram peak heights and pyrosequencing. In the column, ‘Known allelic expression in human’ - P: Paternal, M: Maternal, U: Unknown, =: Biallelic expression. *IGF2R* is polymorphically imprinted in the human population, hence shown as “M/=”.

**Cheong_Supplementary Figure 2**

### Panel A: IGF2R


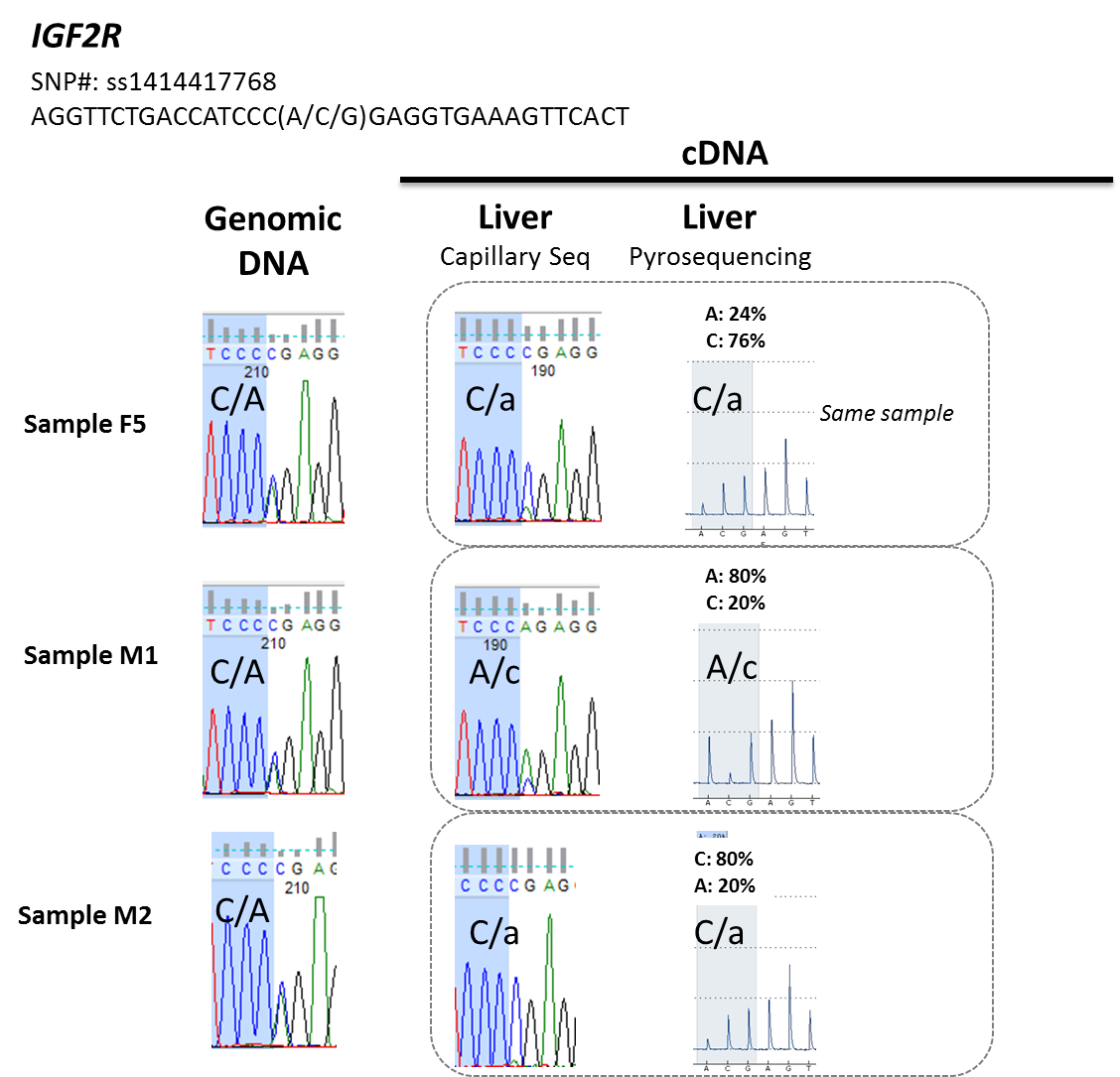


### Panel B: KCNQ1


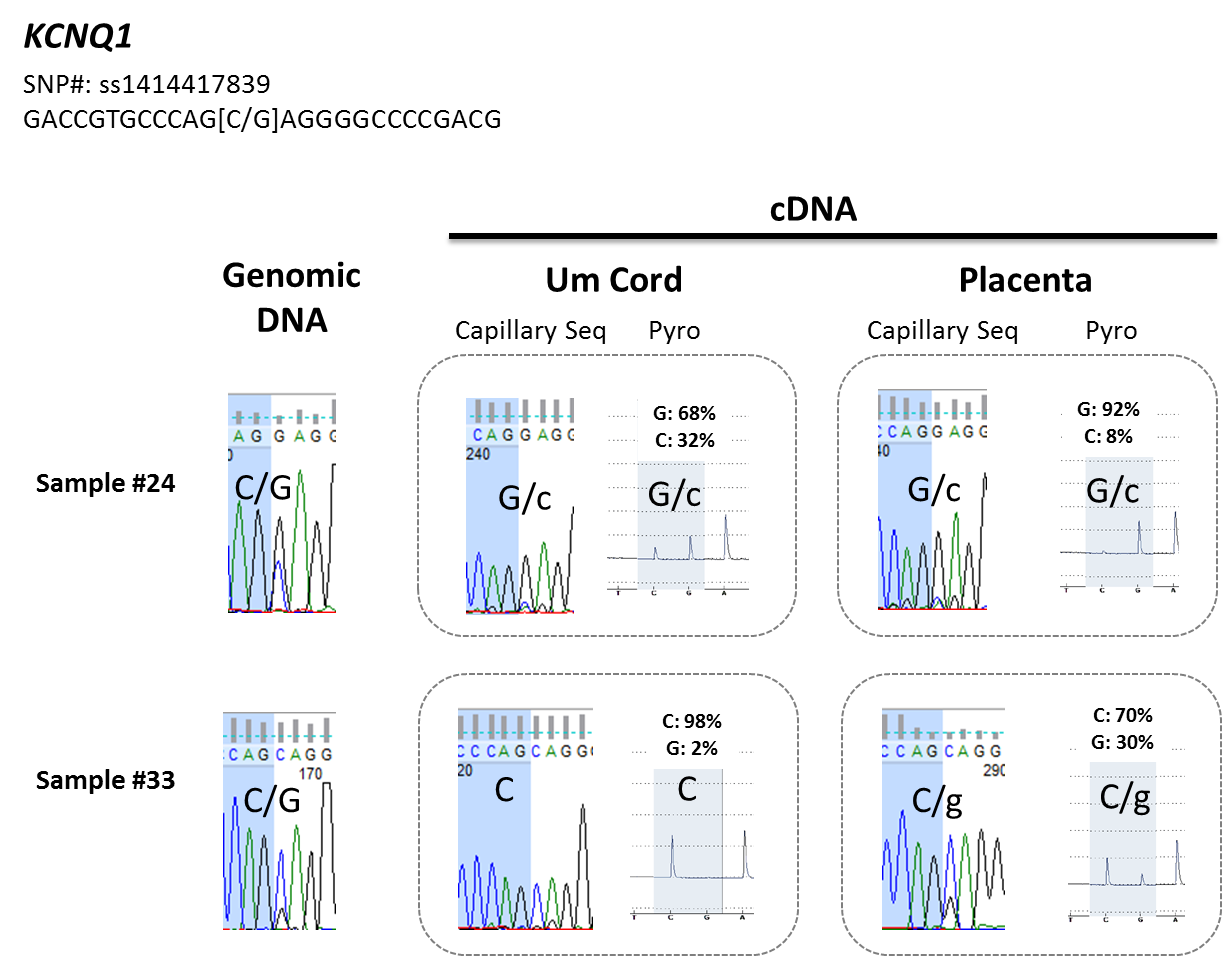


### Panel C: PEG3


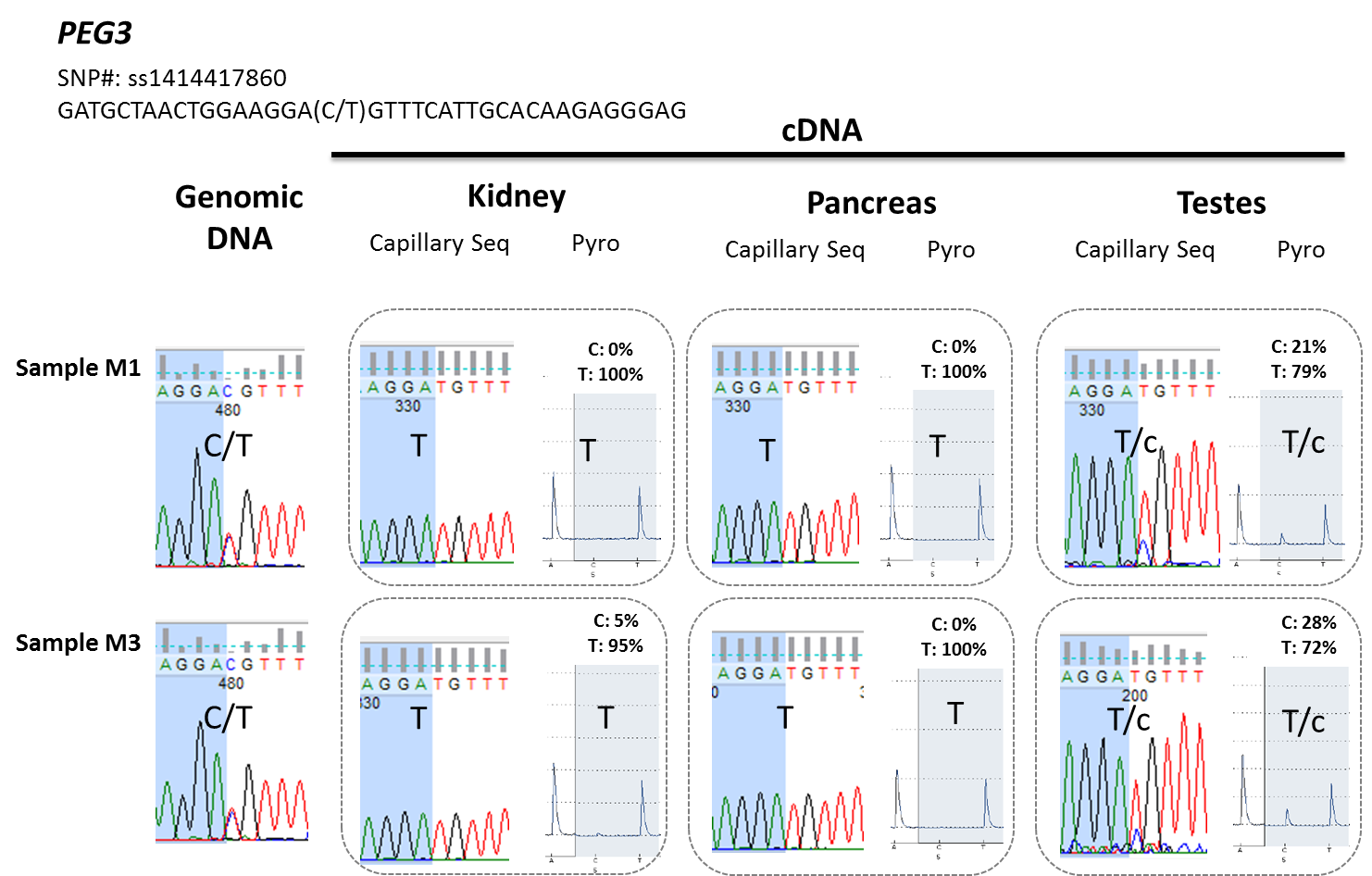


### Panel D: Allelic Expression Is Determined by Pyrosequencing across Tissues and Individuals

| Gene | Tissue/ID | F4 | | F5 | | F6 | | M1 | | M2 | | M3 | | M4 | |
| --- | --- | --- | --- | --- | --- | --- | --- | --- | --- | --- | --- | --- | --- | --- | --- |
|  |  | **%Me** | **Call** | **%Me** | **Call** | **%Me** | **Call** | **%Me** | **Call** | **%Me** | **Call** | **%Me** | **Call** | **%Me** | **Call** |
| *PLAGL1* | Liver |  |  |  |  | A 0: G 100 | G |  |  | A 77: G 23 | A/g |  |  |  |  |
|  | Kidney | A 100 : G 0 | A |  |  | A 4: G 96 | G |  |  | A 100: T 0 | A |  |  |  |  |
|  | Pancreas | A 100 : G 0 | A |  |  | A 0: G 100 | G |  |  | A 100: T 0 | A |  |  |  |  |
| *IGF2* | Liver |  |  |  |  |  |  | A 48: T 52 | T/A | A 48: T 52 | T/A |  |  |  |  |
|  | Kidney |  |  |  |  |  |  | A 13:T 87 | T/a | A 12: T 88 | T/a |  |  |  |  |
|  | Pancreas |  |  |  |  |  |  | A 0: T 100 | T | A 0: T 100 | T |  |  |  |  |
|  | Testes |  |  |  |  |  |  | A 3: T 97 | T |  |  |  |  |  |  |
| *H19* | Liver |  |  | T 4: C 96 | C | T 100: C 0 | T | T 1: C 99 | C |  |  |  |  | T 2: C 98 | C |
|  | Kidney |  |  | T 0 : C 100 | C | T 100: C 0 | T | T 0 : C 100 | C | T 8: C 92 | C/t |  |  |  |  |
|  | Pancreas |  |  | T 7: C 93 | C/t | T 100: C 0 | T |  |  |  |  |  |  |  |  |
|  | Testes |  |  |  |  |  |  | T 7: C 93 | C/t |  |  |  |  |  |  |
| *DLK1* | Liver | C 11: T 89 | T/c | C 86: T 14 | C/t |  |  |  |  |  |  | C 10: T 90 | T/c | C 79: T 21 | C/t |
|  | Kidney | C 0: T 100 | T | C 83: T 17 | C/t |  |  |  |  |  |  | C 0: T 100 | T | C 79: T 21 | C/t |
|  | Pancreas | C 0: T 100 | T | C 100: T 0 | C |  |  |  |  |  |  | C 4: T 96 | T |  |  |
|  | Testes |  |  |  |  |  |  |  |  |  |  | C 45: T55 | T/C |  |  |
| *PEG3* | Liver |  |  |  |  | C 100: T 0 | C | C 0 : T 100 | T |  |  | C 0 : T 100 | T |  |  |
|  | Kidney |  |  |  |  | C 100: T 0 | C | C 0 : T 100 | T |  |  | C 5 : T 95 | T |  |  |
|  | Pancreas |  |  |  |  | C 100: T0 | C | C 0 : T 100 | T |  |  | C 0 : T 100 | T |  |  |
|  | Testes |  |  |  |  |  |  | C 21: T 79 | T/c |  |  | C 28: T 72 | T/c |  |  |
| *IGF2R* | Liver |  |  | A 24: C 76 | C/a | A 80: C 20 | A/c | A 80: C 20 | A/c | A 20: C 80 | C/a |  |  |  |  |
|  | Kidney |  |  | A 16: C 84 | C/a | A 83: C 17 | A/c | A 86: C 14 | A/c | A 23: C 76 | C/a |  |  |  |  |
|  | Pancreas |  |  | A 20: C 80 | C/a | A 81: C 19 | A/c | A 84: C 16 | A/c | A 25: C 75 | C/a |  |  |  |  |
|  | Testes |  |  |  |  |  |  | A 53: C 47 | A/C |  |  |  |  |  |  |
| *KCNQ1* | Liver |  |  | C 58: G 42 | C/G |  |  |  |  | C 50: G 50 | C/G |  |  |  |  |
|  | Kidney |  |  | C 55: G 45 | C/G | C 56: G 44 | C/G |  |  | C 53: G 47 | C/G |  |  |  |  |
|  | Pancreas |  |  | C 53: G 47 | C/G |  |  | C 53: G 47 | C/G | C 55: G 45 | C/G |  |  |  |  |
|  | Testes |  |  |  |  |  |  | C 56: G 44 | C/G |  |  |  |  |  |  |

## Supplementary Figure 2: Pyrosequencing and Capillary Sequencing

To confirm that allelic calls made by capillary sequencing were accurate, we also performed quantitative pyrosequencing across 7 genes in multiple tissues and informative individuals. Data generated from both technology platforms were concordant, and examples for 3 genes are shown in panels A, B & C. In these panels, the allelic calls made by capillary and pyrosequencing are shown as follows: Biallelic (both capitals), Preferential Imprinted Expression (capital for major allele, lower case for minor allele) and Monoallelic (single capital letter). Panel D presents a table view of the pyrosequencing data generated across the imprinted genes tested, percentage expression values are shown for each allele analyzed. Preferential monoallelic expression is noted where the minor allele is expressed at levels between 5 – 30% by pyrosequencing. The samples presented are a subset of subjects analyzed by capillary sequencing.

**Cheong_Supplementary Figure 3**

**
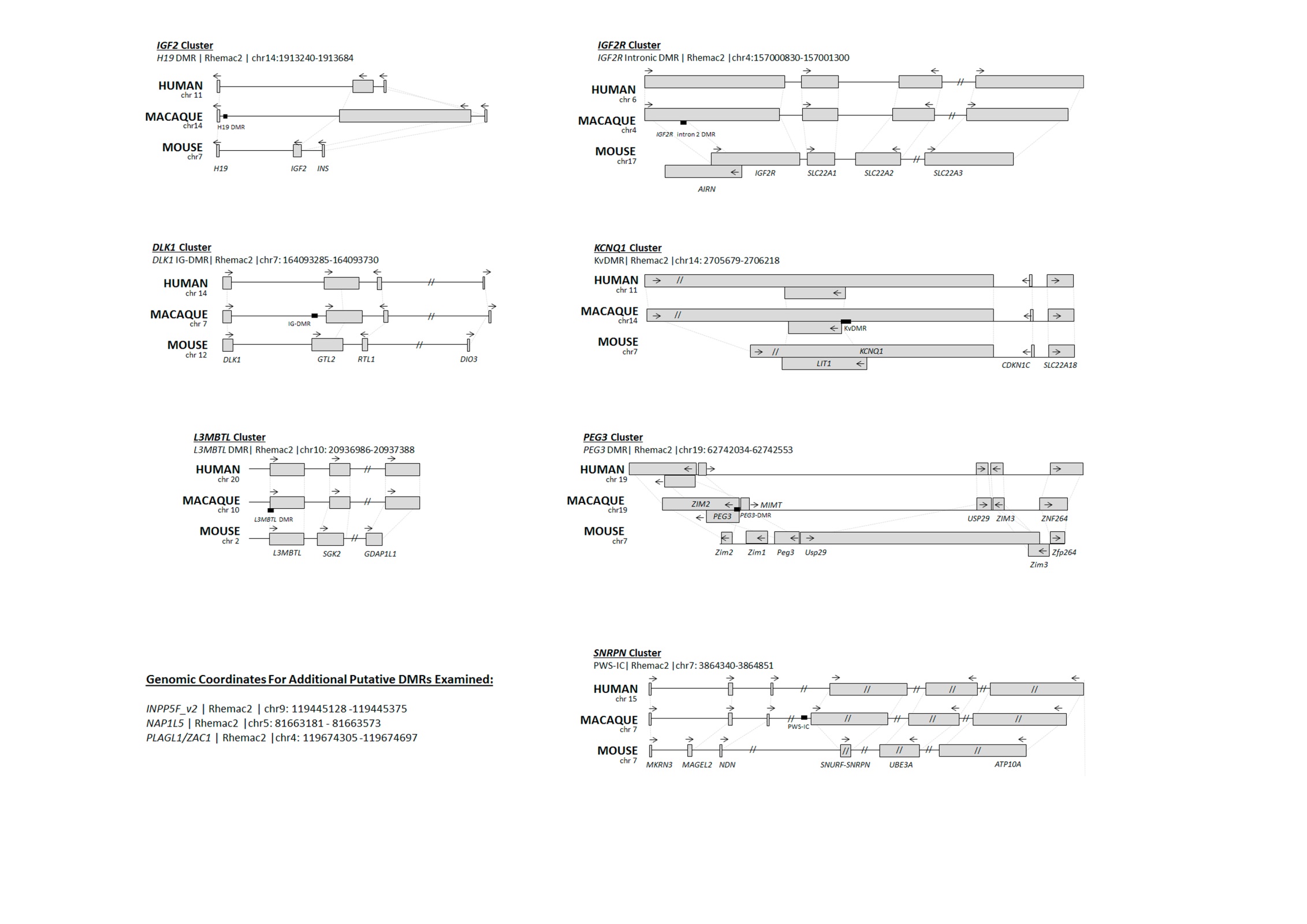
**

## Supplementary Figure 3: Imprinted Gene Cluster Maps and Putative Cynomolgus DMRs

Maps show the conservation in relative gene position and orientation between Human, Macaque and Mouse genomes at the imprinted gene clusters examined. Gene distances are approximately to scale, unless otherwise indicated with a “//”. Genomic coordinates for putative Macaque DMRs examined are indicated. *PLAGL1, NAP1L5* and *INPP5F* were examined as single genes, so no cluster map is shown for these loci.

**Cheong_Supplementary Figure 4**


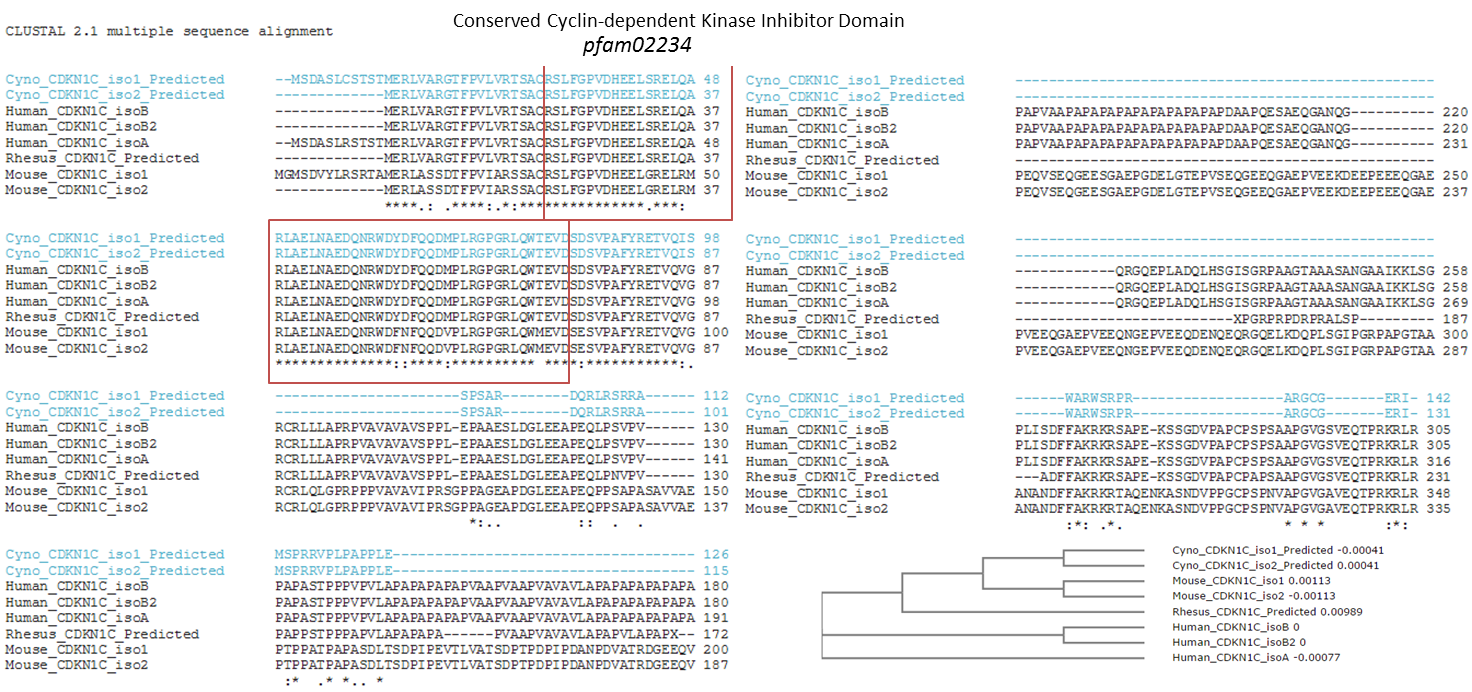


## Supplementary Figure 4. Novel Variant of CDKN1C in Cynomolgus Macaque

The cyclin-dependent kinase inhibitor domain is conserved in CDKN1C proteins across primates and rodents (boxed in red). Downstream of this, there is increasing divergence between CDKN1C protein sequences from multiple species, as also shown by the phylogenetic tree (lower right).

**Cheong_Supplementary Figure 5**


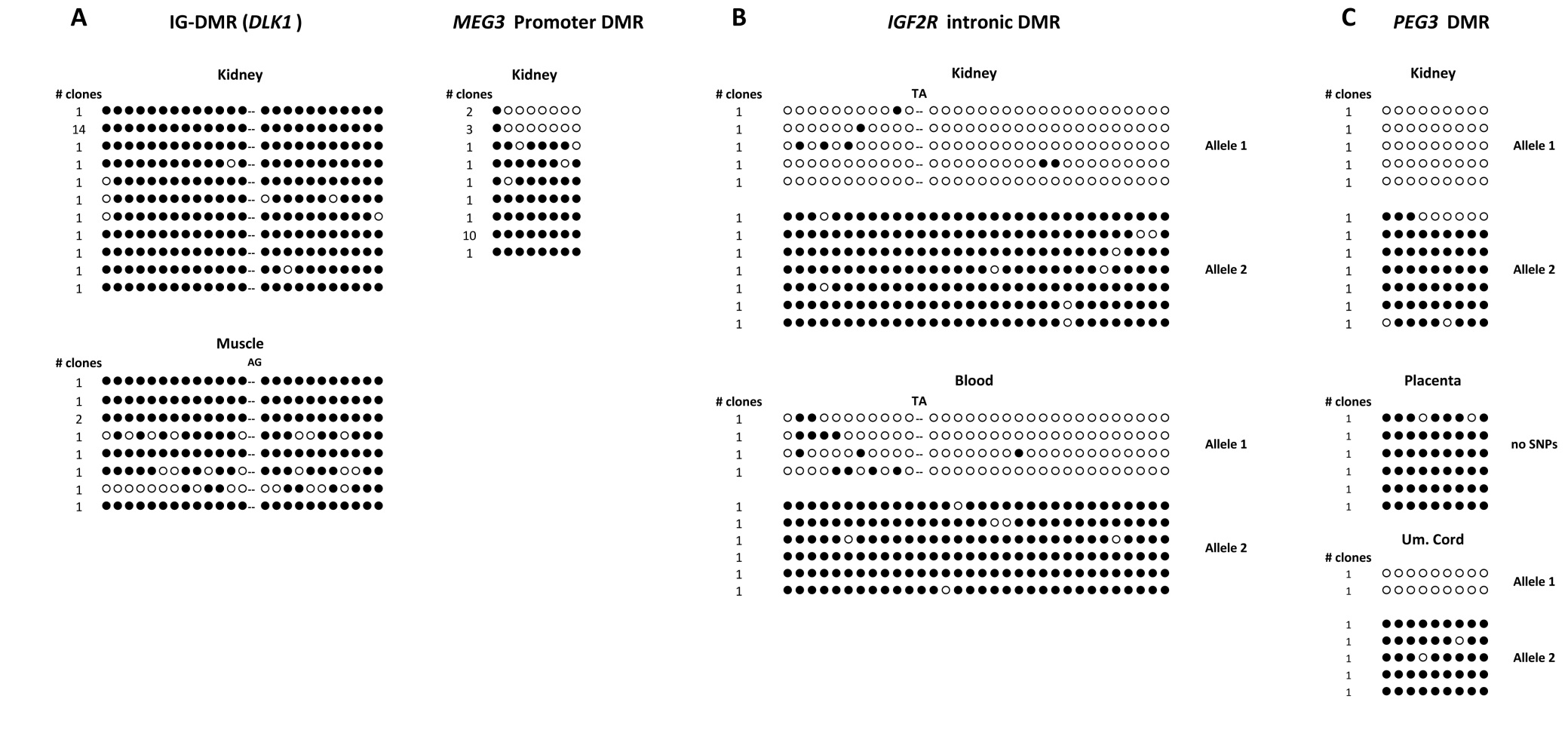


## Supplementary Figure 5. Additional Bisulfite Diagrams of Macaque Samples

DNA methylation in additional tissues/samples for DMR regions shown in Main Figures 1 & 2 – (A) *DLK1* IG-DMR and *MEG3*, where hypermethylation is evident in adult somatic tissues. (B) *IGF2R* DMR, additional somatic tissues (kidney & blood) also showed distinct allele-specific methylation. (C) At the *PEG3* DMR, placenta-specific hypermethylation is seen. This was not observed in somatic tissues or umbilical cord (here, and in main figures)

**Cheong_Supplementary Table 6**

|  | **Human Germline Methylation** | | **Primate Germline Methylation** | | **Mouse Germline Methylation** | |
| --- | --- | --- | --- | --- | --- | --- |
|  | **Oocyte** | **Sperm** | **Oocyte** | **Sperm** | **Oocyte** | **Sperm** |
| ***DLK1*** | **Unmethylated** | **Methylated** | no data | no data | **Unmethylated** | **Methylated** |
|  | *(Geuns et al. 2007a)* | *(Geuns et al. 2007a)* |  |  | *(Hiura et al. 2007)* | *(Hiura et al. 2007)* |
| ***H19*** | **Unmethylated** | **Methylated** | no data | **Methylated** | **Unmethylated** | **Methylated** |
|  | *(Borghol et al. 2006)* | *(Kerjean et al. 2000)* |  | *(Mitalipov et al. 2007)* | *(Tremblay et al. 1995)* | *(Tremblay et al. 1995)* |
| ***IGF2R*** | no data | no data | no data | no data | **Methylated** | **Unmethylated** |
|  |  |  |  |  | *(Lucifero et al. 2002)* | *(Lucifero et al. 2002)* |
| ***INPP5F***  **transcript variant 2** | no data | no data | no data | no data | **Methylated** | **Unmethylated** |
|  |  |  |  |  | *(Wood et al. 2007)* | *(Wood et al. 2007)* |
| ***KCNQ1*** | **Methylated** | **Unmethylated** | no data | no data | **Methylated** | **Unmethylated** |
|  | *(Geuns et al. 2007b)* | *(Geuns et al. 2007b)* |  |  | *(Engemann et al. 2000)* | *(Engemann et al. 2000)* |
| ***L3MBTL1*** | no data | no data | **Methylated** | **Unmethylated** | not imprinted | not imprinted |
|  |  |  | *(Aziz et al. 2013)* | *(Aziz et al. 2013)* |  |  |
| ***PEG3*** | no data | no data | no data | no data | **Methylated** | **Unmethylated** |
|  |  |  |  |  | *(Lucifero et al. 2002)* | *(Lucifero et al. 2002)* |
| ***SNRPN*** | **Methylated** | **Unmethylated** | no data | **Unmethylated** | **Methylated** | **Unmethylated** |
|  | *(Geuns et al. 2003)* | *(El-Maarri et al. 2001; Geuns et al. 2003)* |  | *(Mitalipov et al. 2007)* | *(Lucifero et al. 2002)* | *(Lucifero et al. 2002)* |
|  | **Unmethylated** |  |  |  |  |  |
|  | *(El-Maarri et al. 2001)* |  |  |  |  |  |
| ***PLAGL1*** | no data | no data | no data | no data | **Methylated** | **Unmethylated** |
|  |  |  |  |  | *(Smith et al. 2002)* | *(Lucifero et al. 2002)* |

## Supplementary Table 6: Known Parental Origin of Germline Methylation

Summary of published gamete methylation data for human, primate and mouse at established imprinted loci. Reference details are available at the end of this document.

**Cheong_Supplementary Table 7**

## Supplementary Table 7: Primers for Bisulfite DNA Amplification & Sequencing

|  | |  |  |
| --- | --- | --- | --- |
| **Gene** | **Direction** | **Sequence** | **Tissues** |
| ***H19* DMR** | F | ATTTATGTTTTGGGATGGATGT |  |
|  | R | ATCCTTCCCACAAAATCTCTAA |  |
|  | F | AGAATTTTGGGGTATTTGGTTTG | Oocytes only |
|  | R | CCATATATATTTCTAAAAACTTCCCCTTC | Oocytes only |
| ***DLK1*** | F | GTTAAGAGTTTGTGGATTTGTGAGAAATG |  |
|  | R | CTAAAAATCACCAAAACCCATAAAATCAC |  |
| ***GTL2*** | F | TTGTGTTCGAATTTATTTTGTTTG |  |
|  | R | CCCAAATTCTATAACAAATTACTCTAAC |  |
| **snoDMR** | F | TTTTTTTTTGGAGGGAGAAGG |  |
|  | R | TCTCTCATTCCCCACTTTAAAAAA |  |
| ***KCNQ1* DMR** | F | GATTGGGTAGTAGTAGGGGAAGG |  |
|  | R | CTCACACCCAACCAATACCTC |  |
| ***SNRPN*** | F | TTTGGTTAGTGGTGTGTGTAAA |  |
|  | R | CTTTAAAACCCAAATACCCAA |  |
|  | F | GAGGGTATTGGGATTTTTGTATTG | Oocytes only |
|  | R | CAAACTATCTCTTAAAAAAAACCACC | Oocytes only |
| ***NAP1L5*** | F | TTAGGGTAGTAATAGGAGGAATTTGGTG |  |
|  | R | TAAAACTCCTCAACCATCTAACCAAC |  |
| ***L3MBTL1*** | F | YGAYGTTTTTATAGTTTTTAGGGTTT |  |
|  | R | ACTTTCCCYGAAAACCAATC |  |
| ***PLAGL1*** | F | GTTGGTATAGGAGGTAAGTTAGTTTGG |  |
|  | R | CCCAAACACCTACCCTAC |  |
|  | F | TATTTGTAAAGTATTTAGGATAGTGTTTGG | Oocytes only |
|  | R | CCAAAATAAACTACCTAAACCTAATCACAC | Oocytes only |
| ***IGF2R* intronic DMR** | F | TGTGGTTTTAAAGAGGTTTTGT |  |
|  | R | CTCCTAACAACTTCAAAAAACAA |  |
| ***PEG3*** | F | TTATTTTTGATGGGGGTAGTTG |  |
|  | R | CCCAAACACAAAATAAAACCAC |  |
|  | F | GTTTAGGTGTGGGGTTGTTTATAGG | Oocytes only |
|  | R | CAAATCTCCTAACTCCCCCAAATAC | Oocytes only |
| ***INPP5F* transcript *variant 1*** | F | GGAGGTTATTAGTGGTTATAGTAAGAAGG |  |
|  | R | TTAACTTAAAAAAACTCCATACCAACCC |  |
| ***INPP5F* transcript variant 2** | F | GGAAGTTAGATGTGTTTTTTGGG |  |
|  | R | AAAAAATCTTAAAAATCATAACCACAAC |  |

**Cheong_Supplementary Table 8**

## Supplementary Table 8: Primers for Pyrosequencing of Bisulfite-converted DNA

| **Gene** | **Primer Dir** | **Sequence** |
| --- | --- | --- |
| DLK1 | Forward | CTGTGCTAACAACGGGACCTG |
|  | Reverse | AGGGGCACAGGAGCATTCATA |
|  | Seq | CCTGCGTGAGCCTGG |
| H19 | Forward | AATCGAACACGCTGCACTTTACA |
|  | Reverse | TTCTGGAGCCGATTCCTGAGT |
|  | Seq | AGCCGATTCCTGAGT |
| IGF2 | Forward | GCAAGCGCCCTGTGAGTG |
|  | Reverse | GGGGTGCCTCGAAGTGTTT |
|  | Seq | CCCAAAGAGCCAAAT |
| IGF2R | Forward | CATGGGGATGACCAGGACAG |
|  | Reverse | ACCAGCCCTGCCCTATCGT |
|  | Seq | AGGTTCTGACCATCCC |
| KCNQ1 | Forward | AACGCCCTGCCCACCTAC |
|  | Reverse | CCTTGGCCTCCCCTCTGA |
|  | Seq | GCTGACCGTGCCCAG |
| PEG3 | Forward | AGTCAAGCGACCAGTCACAGA |
|  | Reverse | ATTCCCTTCATAAACCCTCTGC |
|  | Seq | GTCAGATGCTAACTGGAA |
| PLAGL1 | Forward | ATACCTGCCTCTCTGGACCTGTC |
|  | Reverse | GTGCCAGCTGAGAACACATGA |
|  | Seq | ATCTCTGCCCCACAG |

**Cheong_Supplementary Table 9**

## Supplementary Table 9: Primers for Genotyping and RT-PCR

| **Gene** | **Genotyping** | **RT-PCR** | **Direction** | **Sequence** |
| --- | --- | --- | --- | --- |
| *IGF2* | * | * | F | GAAGCCAGCTTGAGACCATC |
|  | * | * | R | ACGAATGGGCAGGTAATTTG |
| *H19* | * | * | F | ATGGTGCTACCCAGCTCAAG |
|  | * |  | R | ATGGAATGCTTGAAGGTTGC |
|  |  | * | R | CACCCAGCCCAGATGAAG |
| *INS* | * | * | F | GGAGCGAGGCTTCTTCTACA |
|  | * | * | R | TGGTTCAAGGGCTTTATTCG |
| *DLK1* | * |  | F | GTGGGAGAAGACAGGCTTTG |
|  | * |  | R | ACAGGAGACTGGGAGGAGGT |
|  |  | * | F | CGACGGCTGGGATGGGAAACT |
|  |  | * | R | GCAGAAATTGCCTGAGAAGCCAG |
| *MEG3 (*also known as *GTL2)* | * |  | F | ACTCCAGCTGCCTGTTTCC |
|  | * |  | R | GTGCCTCCTCCCCAGATTA |
|  |  | * | F | ATGCCTCTCCCTTCCCAGAG |
|  |  | * | R | GCCCGTGCTTTAGAACCGTATC |
| *DIO3* | * | * | F | CCCAGGACTTTGCCTCTACA |
|  | * | * | R | ACACTCACCAAATGGCCTTC |
| *COMMD1 (*also known as *MURR1)* | * |  | F | TTTGGGACCCTGGGTATTTT |
|  | * |  | R | TGAGAACCTCTGCACTGGAA |
|  |  | * | F | GCCTGAGCTGGAGAGTTGAT |
|  |  | * | R | GGAAGATTGAATGCGAGGAT |
| *IGF2R* | * |  | F | GAGCAGAATGGGGTCTCTGA |
|  | * |  | R | CACGTGTGTGCCCTTAAGAA |
|  |  | * | F | TGCTGCAGGAGAAGTTCCAACG |
|  |  | * | R | CCCATCACGAAAACTCTTCCCT |
| *AIRN* |  | * | F | TCAGGAAGTAGGACAGGAAGG |
|  |  | * | R | TTACACACTCACCAGGCCTAC |
| *SLC22A1* | * |  |  | TGCAACAGTTTCATCATCAACA |
|  | * |  |  | TTGGCATTGTGTTTGTTTTAGG |
|  |  | * | F | CGGGAGTGACGCTACTTCTT |
|  |  | * | R | TGGGGGTAGGCAAGTATGAG |
|  |  | * | F | GAACCTCGGAGTGATGGTGT |
| *SLC22A2* | * |  | F | TTGGTGCTTGACTTGACCTG |
|  | * |  | R | GAACAAATGTCCCACCCATC |
|  | * |  | F | TGGCATTCACGAAGACAAGA |
|  | * |  | R | CACATTCATGCTTACCCAGTCT |
|  |  | * | F | GACATTGGTGGCATCATCAC |
|  |  | * | R | CCCATGGTACCCCTGTGT |
| *SLC22A3* | * |  |  | TGGGTTTTCATGAGAAGTTGTG |
|  | * |  |  | CTCTTCCCCAAGACAACTCG |
|  |  | * | F | CTCTTGATCTTACTAACCATTGAG |
|  |  | * | R | CTCTTCCCCAAGACAACTCG |
| *INPP5F* transcript variant 1 | * |  | F | GGATTCCATGGGGGATTAAC |
|  | * |  | R | GCCGAGAAGGTGTCTGTTCT |
|  |  | * | F | CCTCACGAAGACATCATTGG |
|  |  | * | R | GACAAAAACTGTGGGCCTGT |
| *INPP5F* transcript variant 2 | * |  | F | TTGTGCCTTCCCTAATGAGC |
|  | * |  | R | GCTATTCCCAGGACCCTCTC |
|  |  | * | F | GGCTCAAGCAACAATTTTCA |
|  |  | * | R | TGTATGTTGCCAGCAGAAGG |
| *KCNQ1* | * |  | F | CCTGGCCCTCTCTGAAGGAGGC |
|  | * |  | R | TGTTTGGCTGGCTGCTGGGA |
|  | * |  | F | TGACGCAGCTGGACCAGAGGC |
|  | * | * | R | TACCCACCGACCAGGAAGAGGT |
|  |  | * | F | AAGAGCAAGGATCGTGGCAGC |
|  |  | * | R | CCACAGGCCCTCCAAATGGG |
| *CDKN1C* | * |  | F | GCGGTGTTGTTGAAACTGAA |
|  | * |  | R | CTGTCCACTTCGGTCCACTG |
|  | * |  | F | ATTTCTTCGCCAAGCGCAAGA |
|  | * | * | R | CCCAGAGTCCGCGATGGAAATA |
|  |  | * | F | CTCCTTTCCCCTTCTTCTCG |
|  |  | * | F | GTGCCAGCCAGGTCTGAT |
|  |  | * | R | CCTGCTGGAAGTCGTAATCC |
| *SLC22A18* | * |  | F | GGATGGGTCAGGAGAGATGA |
|  | * |  | R | CACTGAAGGACACAGGCAGA |
|  |  | * | F | AGGTGGCCTCCAACTTCC |
|  |  | * | R | GGACCAGAAGGACAAGGACA |
|  |  | * | R | TGGAGGGATTTAGTAGGAGTTTATTG |
| *L3MBTL1* | * |  | F | CCCAGTACACCTGGGTGGCTAGACA |
|  | * |  | R | GCTCAGTGTGGCTCTGCTAGATGCT |
|  |  | * | F | CAGTCGCCAAGTGGACCATC |
|  |  | * | R | GGTAAAGTGATTGGATCTACATGTTCCTCC |
|  |  | * | F | GCACCAGTCCCTCTTCATGT |
|  |  | * | R | TTGTGTTCCCACCATATCAGTC |
| *SGK2* | * |  | F | TAGGAAGCATGGGGCACTCACAG |
|  | * | * | R | CAAAATCCAAACACCAGGAGGCC |
|  |  | * | F | CTCCACCCTTCAACCCAAA |
|  |  | * | R | GGGCAGAAATACAGCCTCTG |
| *GDAP1L1* | * |  | F | GGCAGAAATGCGAGCTGTGG |
|  | * |  | R | CCCCATTCAGAGGTACCGGTTAG |
|  | * |  | F | ATGAGTCCCAGGATGTTTCATCC |
|  | * | * | R | TGGCGTGGATGCAGAGACTTC |
|  |  | * | F | GAGAAGAGGAAGCTGGAGAACGAG |
|  |  | * | F | AACCGGTACCTCTGAATGGG |
| *NAP1L5* | * | * | F | TCCGCAAGATCTCTCTGGAC |
|  | * | * | R | TCCTCCTCGTCATCCTCGTA |
| *PEG3* | * |  | F | GGGATGAGTCTTGGTCCTCA |
|  | * | * | R | TCAAAACGTTTCCCTCCAAC |
|  |  | * | F | CACAATCCAGGACAACATGG |
| *ZIM2* | * |  | F | GCACTAACTCCAGGGCAAAG |
|  | * |  | R | TCAACAGTGATTGGACACAACA |
|  |  | * | F | TATCTCACGCCTGGAAGCGG |
|  |  | * | R | GACTGAAGACTTTGCCACAGTC |
|  |  | * | F | ATCACGTGCAGTGGAGACAG |
|  |  | * | R | CGACTGAAGACTTTGCCACA |
| *USP29* | * |  | F | CAGATTTGGAGCCAGAGGAC |
|  | * |  | R | TGAGAGTCAAGAGGCACCTG |
|  |  | * | F | CTGCCTGCAGTGTGACCAT |
|  |  | * | R | CCATTTTTGGCATCGAGAGTC |
| *ZIM3* | * |  | F | TGAAGCCAGAATGATCACCA |
|  | * |  | R | GGTTTCTCTCCCGTGTGTGT |
|  |  | * | F | ACGTGATCTTGAGGTTGGAGC |
|  |  | * | R | CTTCCACAGCAACGACATTCAA |
| *ZNF264* | * | * | F | ATGGACCAGGAAGGAAGACC |
|  | * |  | R | CCCTTTTGTGTGCTGGATTT |
|  |  | * | R | CTGGTGCCACATGAGGTATG |
|  |  | * | R | ACAACATAGTGCCGGAGAAA |
| *RASGRF1* | * |  | F | ACTTCCCTGGCTCTTTCTCC |
|  | * |  | R | GAGCTTTGTCAGGGGTTCAG |
|  |  | * | F | CAAGCTGCTGAAGCACTACG |
|  |  | * | R | CTCTGGTGATGTTGCCCTTT |
| *MKRN3* | * |  | F | AACTGCCCATTTGGAGACAC |
|  | * |  | R | CAGATGAGCAGCTGCAAGAC |
|  |  | * | F | GCTATTGCCTGTTTTCCCTGT |
|  |  | * | R | TTCTCCTTTCACTCTGCCACA |
| *MAGEL2* | * |  | F | CCCAAAACCACGCTTACATT |
|  | * | * | R | TTCACAAAGCCAGCACAAAG |
|  |  | * | F | TGAGGATGAACCTGACAGCA |
| *NDN* | * | * | F | ATGTGGTACGTGCTGGTCAA |
|  | * | * | R | CCTGGTGAGGATCAGAAAGC |
| *SNRPN* | * |  |  | GGTAGATTGCAGTGCAGCTTT |
|  | * |  |  | AACCCCCATTTACCCAAATC |
|  |  | * | F | GCTCCGTCTACTCTTTGAAGC |
|  |  | * | R | GTTGCTCTGCCAACAGGTG |
| *UBE3A* | * |  | F | TTGCAACAGGGTAAACATGC |
|  | * |  | R | TGGCATCATCATCGTTCACT |
|  | * |  | F | AAAAATCAATGTTGCATGCCTA |
|  | * |  | R | TGTTTGTCGTACTTCGGTCAG |
|  |  | * | F | CCCCAACAACTCCTGCTCT |
|  |  | * | R | TCCAGATATTCAGGACTGTGGA |
|  |  | * | F | CGCATGTACAGTGAACGAAGA |
|  |  | * | R | CCATGGGAAAATGTACATCCA |
| *ATP10A* | * |  | F | AGCTTCCAGAGACCCTCACA |
|  | * |  | R | CCACAGCAATACCTAACCTTTTG |
|  |  | * | F | ATGACACCTGTCGCTGCAC |
|  |  | * | R | ACCTTGGACTCCTCAGGACA |
| *PLAGL1* | * |  | F | CAATACCTGCCTCTCTGGACCTG |
|  | * |  | R | GGCACAATACATGCAGTTCGAGA |
|  | * |  | R | GCAGCTCCTTGGGCAGGTTTAT |
|  |  | * | F | GCAGCCTGACTGTGGCAAAGC |
|  |  | * | R | GATGGAAATGAGGCAGGATGGC |

**Cheong_Supplementary Table 10**

## Supplementary Table 10: List of Identified Cynomolgus SNPs

**Reference: Macaca_fascicularis_5.0 (accession GCF_000364345.1)**

Available through Database of Single Nucleotide Polymorphisms (dbSNP). Bethesda (MD): National Center for Biotechnology Information, National Library of Medicine.

| **Chr** | **Position** | **Ref Allele** | **Alt Allele** | **Gene** | **Ref Seq** | **ssID** |
| --- | --- | --- | --- | --- | --- | --- |
| 4 | 10790382 | G | A | AIRN | NC_022275.1 | ss1414417769 |
| 4 | 10790432 | C | A | AIRN | NC_022275.1 | ss1414417770 |
| 4 | 10790468 | C | T | AIRN | NC_022275.1 | ss1414417771 |
| 4 | 10790661 | A | T | AIRN | NC_022275.1 | ss1414417772 |
| 4 | 10790670 | A | G | AIRN | NC_022275.1 | ss1414417773 |
| 4 | 10693272 | G | A | IGF2R | NC_022275.1 | ss1414417764 |
| 4 | 10693328 | G | C | IGF2R | NC_022275.1 | ss1414417765 |
| 4 | 10693411 | G | A | IGF2R | NC_022275.1 | ss1414417766 |
| 4 | 10693539 | G | A | IGF2R | NC_022275.1 | ss1414417767 |
| 4 | 10693567 | A | G | IGF2R | NC_022275.1 | ss1414417768 |
| 4 | 48472026 | T | C | PLAGL1 | NC_022275.1 | ss1414417874 |
| 4 | 48472205 | A | C | PLAGL1 | NC_022275.1 | ss1414417875 |
| 4 | 48472206 | G | A | PLAGL1 | NC_022275.1 | ss1414417876 |
| 4 | 48472207 | C | A | PLAGL1 | NC_022275.1 | ss1414417877 |
| 4 | 48472212 | C | T | PLAGL1 | NC_022275.1 | ss1536213772 |
| 4 | 48472638 | G | C | PLAGL1 | NC_022275.1 | ss1414417878 |
| 4 | 10636594 | C | A | SLC22A1 | NC_022275.1 | ss1414417759 |
| 4 | 10636597 | T | C | SLC22A1 | NC_022275.1 | ss1414417760 |
| 4 | 10636616 | T | C | SLC22A1 | NC_022275.1 | ss1414417761 |
| 4 | 10636707 | T | C | SLC22A1 | NC_022275.1 | ss1414417762 |
| 4 | 10640420 | G | A | SLC22A1 | NC_022275.1 | ss1414417763 |
| 4 | 10581675 | A | T | SLC22A2 | NC_022275.1 | ss1414417757 |
| 4 | 10581700 | G | A | SLC22A2 | NC_022275.1 | ss1414417758 |
| 4 | 10354358 | C | T | SLC22A3 | NC_022275.1 | ss1414417755 |
| 4 | 10369845 | C | T | SLC22A3 | NC_022275.1 | ss1414417756 |
| 5 | 87839324 | T | A | NAP1L5 | NC_022276.1 | ss1414417774 |
| 5 | 87839372 | C | T | NAP1L5 | NC_022276.1 | ss1414417775 |
| 5 | 87839408 | C | T | NAP1L5 | NC_022276.1 | ss1414417776 |
| 5 | 87839627 | C | T | NAP1L5 | NC_022276.1 | ss1414417777 |
| 7 | 3658170 | T | C | ATP10A | NC_022278.1 | ss1414417793 |
| 7 | 3658171 | T | C | ATP10A | NC_022278.1 | ss1414417794 |
| 7 | 3658205 | A | G | ATP10A | NC_022278.1 | ss1414417795 |
| 7 | 166786348 | G | C | DIO3 | NC_022284.1 | ss1414417798 |
| 7 | 166786627 | G | A | DIO3 | NC_022284.1 | ss1414417799 |
| 7 | 166786641 | A | G | DIO3 | NC_022284.1 | ss1414417800 |
| 7 | 166786710 | A | C | DIO3 | NC_022284.1 | ss1414417801 |
| 7 | 166786786 | G | A | DIO3 | NC_022284.1 | ss1414417802 |
| 7 | 165945992 | C | T | DLK1 | NC_022284.1 | ss1414417796 |
| 7 | 166028190 | C | A | DLK1 CG6 | NC_022284.1 | ss1414417879 |
| 7 | 1950762 | G | A | MAGEL2 | NC_022278.1 | ss1414417780 |
| 7 | 1950801 | G | T | MAGEL2 | NC_022278.1 | ss1414417781 |
| 7 | 1950829 | A | C | MAGEL2 | NC_022278.1 | ss1414417782 |
| 7 | 166078426 | C | T | MEG3 | NC_022284.1 | ss1414417797 |
| 7 | 1871919 | T | C | MKRN3 | NC_022278.1 | ss1414417778 |
| 7 | 1872057 | C | T | MKRN3 | NC_022278.1 | ss1414417779 |
| 7 | 1992100 | C | T | NDN | NC_022278.1 | ss1414417783 |
| 7 | 1992475 | G | A | NDN | NC_022278.1 | ss1414417784 |
| 7 | 1992559 | G | A | NDN | NC_022278.1 | ss1414417785 |
| 7 | 1992574 | G | C | NDN | NC_022278.1 | ss1414417786 |
| 7 | 2956185 | T | C | SNRPN | NC_022278.1 | ss1414417787 |
| 7 | 2956265 | G | A | SNRPN | NC_022278.1 | ss1414417788 |
| 7 | 2957175 | A | C | SNRPN | NC_022278.1 | ss1414417789 |
| 7 | 2957248 | G | A | SNRPN | NC_022278.1 | ss1414417790 |
| 7 | 3373292 | A | G | UBE3A | NC_022278.1 | ss1414417791 |
| 7 | 3373403 | G | C | UBE3A | NC_022278.1 | ss1414417792 |
| 9 | 119124377 | C | T | INPP5F | NC_022280.1 | ss1414417805 |
| 9 | 119124674 | G | A | INPP5F | NC_022280.1 | ss1414417807 |
| 9 | 119124728 | T | C | INPP5F | NC_022280.1 | ss1414417809 |
| 9 | 119124944 | A | C | INPP5F | NC_022280.1 | ss1414417811 |
| 9 | 119119772 | A | G | INPP5F-v2 | NC_022280.1 | ss1414417803 |
| 9 | 119119790 | C | T | INPP5F-v2 | NC_022280.1 | ss1414417804 |
| 9 | 119124599 | G | A | INPP5F-v2 | NC_022280.1 | ss1414417806 |
| 9 | 119124674 | G | A | INPP5F-v2 | NC_022280.1 | ss1414417807 |
| 9 | 119124728 | T | C | INPP5F-v2 | NC_022280.1 | ss1414417809 |
| 10 | 75987131 | T | C | GDAP1L1 | NC_022281.1 | ss1414417827 |
| 10 | 75987192 | C | T | GDAP1L1 | NC_022281.1 | ss1414417828 |
| 10 | 75987409 | G | T | GDAP1L1 | NC_022281.1 | ss1414417829 |
| 10 | 75227729 | G | A | L3MBTL1 | NC_022281.1 | ss1414417812 |
| 10 | 75227742 | C | T | L3MBTL1 | NC_022281.1 | ss1414417813 |
| 10 | 75227850 | G | A | L3MBTL1 | NC_022281.1 | ss1414417814 |
| 10 | 75227989 | G | C | L3MBTL1 | NC_022281.1 | ss1414417815 |
| 10 | 75228152 | C | T | L3MBTL1 | NC_022281.1 | ss1414417816 |
| 10 | 75228455 | C | A | L3MBTL1 | NC_022281.1 | ss1414417817 |
| 10 | 75228514 | A | G | L3MBTL1 | NC_022281.1 | ss1414417818 |
| 10 | 75228522 | T | C | L3MBTL1 | NC_022281.1 | ss1414417819 |
| 10 | 75271725 | C | T | SGK2 | NC_022281.1 | ss1414417820 |
| 10 | 75271925 | A | T | SGK2 | NC_022281.1 | ss1414417821 |
| 10 | 75271927 | G | A | SGK2 | NC_022281.1 | ss1414417822 |
| 10 | 75271947 | C | T | SGK2 | NC_022281.1 | ss1414417823 |
| 10 | 75272039 | G | A | SGK2 | NC_022281.1 | ss1414417824 |
| 10 | 75272073 | G | A | SGK2 | NC_022281.1 | ss1414417825 |
| 10 | 75272085 | T | A | SGK2 | NC_022281.1 | ss1414417826 |
| 13 | 47924764 | G | A | COMMD1 | NC_022284.1 | ss1414417830 |
| 13 | 47924765 | C | T | COMMD1 | NC_022284.1 | ss1414417831 |
| 13 | 47924814 | C | T | COMMD1 | NC_022284.1 | ss1414417832 |
| 14 | 2847097 | C | G | CDKN1C | NC_022285.1 | ss1414417844 |
| 14 | 2847308 | A | C | CDKN1C | NC_022285.1 | ss1414417845 |
| 14 | 2847352 | A | C | CDKN1C | NC_022285.1 | ss1414417846 |
| 14 | 1965708 | G | A | H19 | NC_022285.1 | ss1414417833 |
| 14 | 1966174 | A | G | H19 | NC_022285.1 | ss1414417834 |
| 14 | 1972491 | C | T | H19 DMR | NC_022285.1 | ss1414417880 |
| 14 | 1972492 | G | A | H19 DMR | NC_022285.1 | ss1414417881 |
| 14 | 1972926 | C | G | H19 DMR | NC_022285.1 | ss1414417882 |
| 14 | 2089839 | G | C | IGF2 | NC_022285.1 | ss1414417835 |
| 14 | 2111382 | C | T | INS | NC_022285.1 | ss1414417836 |
| 14 | 2111387 | C | T | INS | NC_022285.1 | ss1414417837 |
| 14 | 2808350 | G | A | KCNQ1 | NC_022285.1 | ss1414417838 |
| 14 | 2808454 | G | C | KCNQ1 | NC_022285.1 | ss1414417839 |
| 14 | 2808540 | T | C | KCNQ1 | NC_022285.1 | ss1414417840 |
| 14 | 2808560 | C | T | KCNQ1 | NC_022285.1 | ss1414417841 |
| 14 | 2808587 | G | A | KCNQ1 | NC_022285.1 | ss1414417842 |
| 14 | 2808596 | G | A | KCNQ1 | NC_022285.1 | ss1414417843 |
| 14 | 2888638 | A | G | SLC22A18 | NC_022285.1 | ss1414417847 |
| 14 | 2888699 | A | G | SLC22A18 | NC_022285.1 | ss1414417848 |
| 14 | 2888770 | G | A | SLC22A18 | NC_022285.1 | ss1414417849 |
| 19 | 57532401 | C | T | PEG3 | NC_022290.1 | ss1414417853 |
| 19 | 57532464 | C | T | PEG3 | NC_022290.1 | ss1414417854 |
| 19 | 57532530 | C | A | PEG3 | NC_022290.1 | ss1414417855 |
| 19 | 57532533 | T | C | PEG3 | NC_022290.1 | ss1414417856 |
| 19 | 57532629 | A | G | PEG3 | NC_022290.1 | ss1414417857 |
| 19 | 57532650 | C | T | PEG3 | NC_022290.1 | ss1414417858 |
| 19 | 57532692 | T | C | PEG3 | NC_022290.1 | ss1414417859 |
| 19 | 57532791 | G | A | PEG3 | NC_022290.1 | ss1414417860 |
| 19 | 57805125 | G | C | USP29 | NC_022290.1 | ss1414417861 |
| 19 | 57805202 | G | C | USP29 | NC_022290.1 | ss1414417862 |
| 19 | 57805218 | A | G | USP29 | NC_022290.1 | ss1414417863 |
| 19 | 57805273 | G | A | USP29 | NC_022290.1 | ss1414417864 |
| 19 | 57805487 | C | T | USP29 | NC_022290.1 | ss1414417865 |
| 19 | 57482406 | A | G | ZIM2 | NC_022290.1 | ss1414417850 |
| 19 | 57482689 | G | T | ZIM2 | NC_022290.1 | ss1414417851 |
| 19 | 57482881 | G | A | ZIM2 | NC_022290.1 | ss1414417852 |
| 19 | 57811184 | A | G | ZIM3 | NC_022290.1 | ss1414417866 |
| 19 | 57811206 | C | T | ZIM3 | NC_022290.1 | ss1414417867 |
| 19 | 57811463 | A | G | ZIM3 | NC_022290.1 | ss1414417868 |
| 19 | 57811504 | G | A | ZIM3 | NC_022290.1 | ss1414417869 |
| 19 | 57873671 | G | A | ZNF264 | NC_022290.1 | ss1414417870 |
| 19 | 57873684 | C | A | ZNF264 | NC_022290.1 | ss1414417871 |
| 19 | 57874183 | A | G | ZNF264 | NC_022290.1 | ss1414417872 |
| 19 | 57874189 | G | A | ZNF264 | NC_022290.1 | ss1414417873 |

**REFERENCES**

Aziz A, Baxter EJ, Edwards C, Cheong CY, Ito M, Bench A, Kelley R, Silber Y, Beer PA, Chng K et al. 2013. Cooperativity of imprinted genes inactivated by acquired chromosome 20q deletions. *The Journal of clinical investigation* **123**(5): 2169-2182.

Borghol N, Lornage J, Blachere T, Sophie Garret A, Lefevre A. 2006. Epigenetic status of the H19 locus in human oocytes following in vitro maturation. *Genomics* **87**(3): 417-426.

El-Maarri O, Buiting K, Peery EG, Kroisel PM, Balaban B, Wagner K, Urman B, Heyd J, Lich C, Brannan CI et al. 2001. Maternal methylation imprints on human chromosome 15 are established during or after fertilization. *Nat Genet* **27**(3): 341-344.

Engemann S, Strodicke M, Paulsen M, Franck O, Reinhardt R, Lane N, Reik W, Walter J. 2000. Sequence and functional comparison in the Beckwith-Wiedemann region: implications for a novel imprinting centre and extended imprinting. *Hum Mol Genet* **9**(18): 2691-2706.

Geuns E, De Rycke M, Van Steirteghem A, Liebaers I. 2003. Methylation imprints of the imprint control region of the SNRPN-gene in human gametes and preimplantation embryos. *Hum Mol Genet* **12**(22): 2873-2879.

Geuns E, De Temmerman N, Hilven P, Van Steirteghem A, Liebaers I, De Rycke M. 2007a. Methylation analysis of the intergenic differentially methylated region of DLK1-GTL2 in human. *Eur J Hum Genet* **15**(3): 352-361.

Geuns E, Hilven P, Van Steirteghem A, Liebaers I, De Rycke M. 2007b. Methylation analysis of KvDMR1 in human oocytes. *J Med Genet* **44**(2): 144-147.

Hiura H, Komiyama J, Shirai M, Obata Y, Ogawa H, Kono T. 2007. DNA methylation imprints on the IG-DMR of the Dlk1-Gtl2 domain in mouse male germline. *FEBS Lett* **581**(7): 1255-1260.

Kerjean A, Dupont JM, Vasseur C, Le Tessier D, Cuisset L, Paldi A, Jouannet P, Jeanpierre M. 2000. Establishment of the paternal methylation imprint of the human H19 and MEST/PEG1 genes during spermatogenesis. *Hum Mol Genet* **9**(14): 2183-2187.

Lucifero D, Mertineit C, Clarke HJ, Bestor TH, Trasler JM. 2002. Methylation dynamics of imprinted genes in mouse germ cells. *Genomics* **79**(4): 530-538.

Mitalipov S, Clepper L, Sritanaudomchai H, Fujimoto A, Wolf D. 2007. Methylation status of imprinting centers for H19/IGF2 and SNURF/SNRPN in primate embryonic stem cells. *Stem Cells* **25**(3): 581-588.

Smith RJ, Arnaud P, Konfortova G, Dean WL, Beechey CV, Kelsey G. 2002. The mouse Zac1 locus: basis for imprinting and comparison with human ZAC. *Gene* **292**(1-2): 101-112.

Tremblay KD, Saam JR, Ingram RS, Tilghman SM, Bartolomei MS. 1995. A paternal-specific methylation imprint marks the alleles of the mouse H19 gene. *Nat Genet* **9**(4): 407-413.

Wood AJ, Bourc'his D, Bestor TH, Oakey RJ. 2007. Allele-specific demethylation at an imprinted mammalian promoter. *Nucleic Acids Res* **35**(20): 7031-7039.
