# Supplementary material for: Germline and somatic imprinting in the nonhuman primate highlights species differences in oocyte methylation
Source: Genome Res. 2015 May;25(5):611–23. doi: 10.1101/gr.183301.114 (PMC4417110; doi:10.1101/gr.183301.114)
Supplement: Supplemental Material [file supp_gr.183301.114_Supplemental_Material.docx]

## SUPPLEMENTARY MATERIAL

**Supplementary File 1**

**Supplementary Table 1:** Allelic Expression by NHP Tissue and Gene

**Supplementary Figure 2:** Pyrosequencing and Capillary Sequencing Data

Supplementary Figure 3: Imprinted Gene Cluster Maps and Putative Cynomolgus DMRs

**Supplementary Figure 4:** Novel Variant of CDKN1C in Cynomolgus Macaque

**Supplementary Figure 5:** Additional Bisulfite Diagrams of Macaque Samples

**Supplementary Table 6:** Known Parental Origin of Germline Methylation

Supplementary Table 7: Primers for Bisulfite DNA Amplification & Sequencing

Supplementary Table 8: Primers for Pyrosequencing of Bisulfite-converted DNA

**Supplementary Table 9:** Primers for Genotyping and RT-PCR

**Supplementary Table 10:** List of Identified Cynomolgus SNPs

**Supplementary File 2: Allele Specific Expression - Pyrosequencing Data**
